# Nuciferine modulates the gut microbiota and prevents obesity in high-fat diet-fed rats

**DOI:** 10.1038/s12276-020-00534-2

**Published:** 2020-12-01

**Authors:** Yu Wang, Weifan Yao, Bo Li, Shiyun Qian, Binbin Wei, Shiqiang Gong, Jing Wang, Mingyan Liu, Minjie Wei

**Affiliations:** 1 School of Pharmacy, China Medical University, Shenyang, 110122 China; 2 Liaoning Key Laboratory of Molecular Targeted Anti-Tumor Drug Development and Evaluation; Liaoning Cancer Immune Peptide Drug Engineering Technology Research Center; Key Laboratory of Precision Diagnosis and Treatment of Gastrointestinal Tumors, Ministry of Education, China Medical University, Shenyang, 110122 China

**Keywords:** Obesity, Drug development

## Abstract

Gut microbiota dysbiosis has a significant role in the pathogenesis of metabolic diseases, including obesity. Nuciferine (NUC) is a main bioactive component in the lotus leaf that has been used as food in China since ancient times. Here, we examined whether the anti-obesity effects of NUC are related to modulations in the gut microbiota. Using an obese rat model fed a HFD for 8 weeks, we show that NUC supplementation of HFD rats prevents weight gain, reduces fat accumulation, and ameliorates lipid metabolic disorders. Furthermore, 16S rRNA gene sequencing of the fecal microbiota suggested that NUC changed the diversity and composition of the gut microbiota in HFD-fed rats. In particular, NUC decreased the ratio of the phyla Firmicutes/Bacteroidetes, the relative abundance of the LPS-producing genus *Desulfovibrio* and bacteria involved in lipid metabolism, whereas it increased the relative abundance of SCFA-producing bacteria in HFD-fed rats. Predicted functional analysis of microbial communities showed that NUC modified genes involved in LPS biosynthesis and lipid metabolism. In addition, serum metabolomics analysis revealed that NUC effectively improved HFD-induced disorders of endogenous metabolism, especially lipid metabolism. Notably, NUC promoted SCFA production and enhanced intestinal integrity, leading to lower blood endotoxemia to reduce inflammation in HFD-fed rats. Together, the anti-obesity effects of NUC may be related to modulations in the composition and potential function of gut microbiota, improvement in intestinal barrier integrity and prevention of chronic low-grade inflammation. This research may provide support for the application of NUC in the prevention and treatment of obesity.

## Obesity: Getting leaner on lotus leaves

A natural compound found in lotus leaf could treat and prevent obesity by stabilizing disrupted gut microbiota and reducing the associated chronic. Obesity is a considerable health burden worldwide, yet treatment options are limited. The composition of an individual’s gut microbiota influences the development of obesity; an imbalance in the ratio of two bacterial species in particular can accelerate the disease. Mingyan Liu and Minjie Wei at the China Medical University, Shenyang, and co-workers demonstrated that nuciferine, a bioactive component of lotus leaf, reduced weight gain and fat accumulation in rats fed a high-fat diet. Nuciferine changed the diversity and composition of the rats’ gut microbiota, and modified the expression of genes involved in processes such as lipid metabolism. Nuciferine also enhanced intestinal integrity, and reduced chronic low-level inflammation.

## Introduction

Obesity is a global epidemic associated with poor quality of life and reduced average life expectancy^1^. The characteristics of obesity include excess fat accumulation in the adipose tissue and organs in the body, a significant increase in plasma lipid levels, and chronic, low-grade inflammation. Excessive body weight increases the risk of many chronic diseases, particularly type 2 diabetes, cardiovascular disease, fatty liver disease, and certain types of cancer^2^. In 2015, a total of 107.7 million children and 603.7 million adults were obese. Since 1980, the prevalence of obesity has doubled in more than 70 countries and has continuously increased in most other countries^3^. Therefore, the prevention and treatment of obesity has become a major public health issue, and novel treatment strategies would be highly beneficial. Unfortunately, available anti-obesity drugs are of limited efficacy and are associated with serious adverse effects^4^.

Recently, a rapidly expanding area of research has shown that gut microbiota dysbiosis is associated with obesity and obesity-induced metabolic disorders in animals and humans, mainly by modulating nutrient acquisition, energy regulation, and fat storage^5,6^. It has been reported that a disparity in gut microbial composition exists between obese and lean subjects, characterized by an increased ratio of the major phyla Firmicutes/Bacteroidetes^7^. Moreover, after high-fat diet (HFD) feeding for 4 weeks, the intestinal ecosystem increases gut permeability, allowing the gut microbiota-derived endotoxin LPS into the bloodstream and leading to chronic inflammation and eventually metabolic disorders in obese mice^8^. In addition, SCFAs such as acetic, propionic and butyric acids, the main products of dietary fiber metabolized by the gut microbiota, were decreased in obese mice^9^. These SCFAs play key functional roles in the pathophysiology of obesity by improving insulin sensitivity and glucose and lipid homeostasis, decreasing inflammation, and enhancing mucosal barrier function^10,11^. Thus, the gut microbiota is a potential nutritional and pharmacological target in the management of obesity and obesity-related disorders.

The active ingredients isolated from traditional Chinese medicine (TCM) have been demonstrated to ameliorate host obesity associated with HFD by modulating gut microbiota^12^. For example, berberine, a major pharmacological component of *Coptis chinensis*, prevented the development of obesity and insulin resistance in HFD-fed rats through structural modulation of the gut microbiota^13^. In addition, hydroxysafflor yellow A, an effective water-soluble monomer of safflower, reduced obesity, enhanced intestinal integrity, and increased SCFA production in HFD-fed mice by modulating gut microbiota^14^. Thus, it is of great value to explore active components that have the ability to reduce obesity via gut microbiota.

**Fig. 1:**
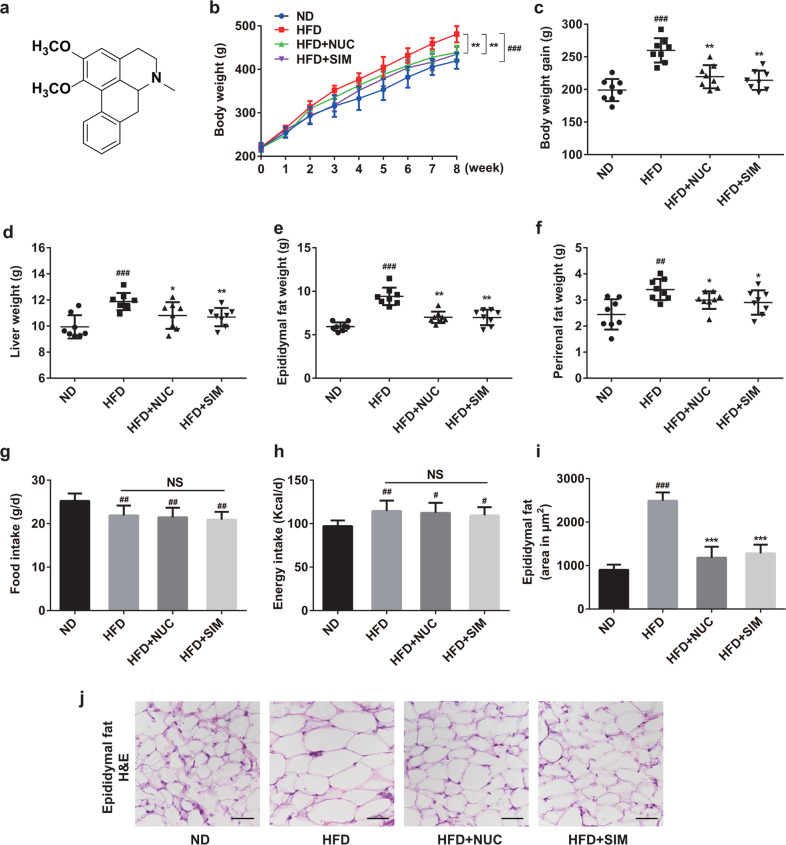
NUC reduces body weight and fat accumulation in HFD-fed rats. The chemical structure of NUC (**a**) and the effects of NUC treatment on body weight (**b**), body weight gain (**c**), liver weight (**d**), epididymal fat weight (**e**), and perirenal fat weight (**f**) are shown. Food intake (**g**) and energy intake (**h**) were monitored. Energy intake was determined based on the calorie intake from consumed food. Epididymal fat size (**j**) was demonstrated using H&E staining (magnification = 200×, scale bar, 50 μm). Mean epididymal fat size (**i**) was estimated using ImageJ software. Values are presented as the mean ± SD (*n* = 8 per group). ^#^*P* < 0.05, ^##^*P* < 0.01, ^###^*P* < 0.001 vs ND, *P < 0.05, **P < 0.01, ***P < 0.001 vs HFD. NS not significant.

*Nelumbo nucifera* Gaertn. (Lotus) has primarily been used as food in China since ancient times. The seeds, plumule, and leaves can be used in TCM for the treatment of fever, diarrhea and bleeding^15^. Nuciferine (NUC, Fig. 1a), an aromatic ring-containing alkaloid, is a main bioactive component obtained from the lotus leaf and has been reported to possess extensive pharmacological activities, including but not limited to anticancer, anti-inflammation, antioxidation, and vasorelaxant effects^15^. Notably, NUC treatment alleviated dyslipidemia as well as liver steatosis and injury by inhibiting the expression of hepatic genes involved in lipid metabolism^16,17^. Moreover, NUC was shown to improve the lipid profile and attenuate hepatic steatosis by activating the PPARα/PGC1α pathway in HFD/streptozotocin-induced diabetic mice^18^. Nevertheless, it is still difficult to fully explain the effects of NUC given its poor oral bioavailability^19^. However, to our knowledge, the effect of NUC on gut microbiota in obesity in particular is unknown. Therefore, the aim of the present study was to investigate the regulation of gut microbiota structure by NUC in HFD-fed rats and identify the relationship between gut microbiota and the anti-obesity effect of NUC.

In the present study, we examined the effects of NUC supplementation in HFD-induced obese rats and investigated the composition of gut microbiota by applying 16S rRNA sequencing. Then, metabolomic profiling of serum samples was performed by ultra-high-performance liquid chromatography coupled with quadrupole time-of-flight mass spectrometry (UPLC/Q-TOF-MS). Moreover, we performed an integrative analysis of the relations between specific gut microbiota and disease biomarkers to understand the potential anti-obesity mechanisms of NUC. This research may provide support for the application of NUC in the prevention and treatment of obesity.

## Materials and methods

### Materials and diet

NUC (purity > 98%) was obtained from Xi’an Tianbao Biological Technology Co., Ltd. (Shaanxi, China). Simvastatin (SIM) was purchased from MSD Pharmaceutical Co., Ltd. (Hangzhou, China). NUC and SIM were each suspended in a 0.3% sodium carboxyl methyl cellulose (CMC-Na) aqueous solution. Normal diet (ND, 10% calories from fat, D12450J) and HFD (HFD, 60% calories from fat, D12492) were purchased from Research Diets Inc. (New Brunswick, NJ, USA)^14^.

### Animals and treatment

All animal experiments were approved and performed in accordance with the Standard Medical Laboratory Animals Care and Use Protocols (Ministry of Health PR China, 1998) and the Laboratory Animal Ethical Standards of China Medical University (CMU2019236). Male Sprague-Dawley rats (180–200 g) were purchased from Liaoning Changsheng Biotechnology Co., Ltd. (Liaoning, China). All rats were maintained in a controlled environment (a 12-h light/dark cycle, 22 ± 2 °C, and 60 ± 10% humidity) with free access to food and water. After 1 week of acclimation, the rats were randomly divided into five groups (ND, ND + NUC, HFD, HFD + NUC, and HFD + SIM, *n* = 8 per group). The rats in the ND + NUC, HFD + NUC and HFD + SIM groups were fed ND or HFD and intragastrically received NUC (10 mg/kg) and SIM (10 mg/kg) for 8 weeks^16,19^. The rats in the ND and HFD groups were fed ND or HFD and intragastrically received the same volume of 0.3% CMC-Na aqueous solution for 8 weeks. Food intake was monitored every day and used to calculate the energy intake of each rat. The body weight was recorded weekly. At the end of the experiment, rats were euthanized, and blood samples were collected from the abdominal aorta. The serum was extracted by centrifugation of the blood samples at 1500 × *g* for 15 min at 4 °C and stored at −80 °C for subsequent analysis. Epididymal and perirenal fat, liver, colon, and feces were immediately excised, weighed, snap-frozen in liquid nitrogen, and stored at −80 °C for subsequent analysis.

### Biochemical analysis

The serum levels of triglyceride (TG), total cholesterol (TC), high-density lipoprotein cholesterol (HDL-C), low-density lipoprotein cholesterol (LDL-C), alanine aminotransferase (ALT), and alkaline phosphatase (ALP) were measured by using the Mindray BS-200 automatic biochemical analyzer with the corresponding commercial kits (Mindray, Shenzhen, China). Tumor necrosis factor-α (TNF-α), IL-1β, and IL-6 protein levels were examined with commercial ELISA kits (Chenglin Biotechnology, Beijing, China). Serum LPS quantification was determined using a commercial kit (Chenglin Biotechnology, Beijing, China) based on the manufacturer’s instructions.

### Histological analysis

Epididymal fat and liver were fixed in 4% paraformaldehyde, embedded in paraffin, sectioned at 4 μm and stained with hematoxylin and eosin (H&E). For Oil Red O staining, the livers were fixed and embedded in OCT cryo-embedding compound. Frozen sections (8-μm thick) were cut and stained with Oil Red O (Sigma-Aldrich, USA). Stained sections were examined by light microscopy (ECLIPSE 80i; Nikon, Tokyo, Japan), and adipocyte size was analyzed using ImageJ software.

### Quantitative real-time PCR analysis

Total RNA was extracted from liver and colon tissues using TRIzol reagent (Beyotime Biotechnology, Beijing, China). Single-stranded cDNA was synthesized using the Transcriptor First Strand cDNA Synthesis Kit (TransGen Biotechnology, Beijing, China). Quantitative analysis of the expression of fatty acid synthase (FAS), sterol regulatory element-binding protein-1 (SREBP-1), peroxisome proliferator-activated receptors-ɑ/γ (PPARɑ/γ), TNF-α, IL-1β, IL-6, IL-10, zonula occludens (ZO)-1, occludin, and glyceraldehyde-3-phosphate dehydrogenase (GAPDH) mRNA was performed using RT-PCR with 96‐well optical reaction plates using the QuantStudio 3 Real-Time PCR System (Thermo Fisher Scientific Inc., USA). The primers used are listed in the Supplementary Information Table S1. The gene expression was normalized to the housekeeping gene GAPDH, and the expression relative to the control group was calculated according to the 2^−ΔΔCt^ method.

### Western blotting

The colon tissues were homogenized in RIPA buffer (Beyotime Biotechnology, Beijing, China) containing 0.1% protease inhibitor PMSF (Sigma-Aldrich, USA). Tissue homogenates were centrifuged at 12,000 × *g* for 5 min at 4 °C, and the supernatants were collected. The protein concentration was measured using a BCA kit (Beyotime Biotechnology, Beijing, China). Proteins (60 μg) were separated by SDS-PAGE and then transferred to a PVDF membrane (Millipore, Billerica, MA, USA). The membrane was incubated with PBS containing 0.1% Tween-20 (PBST) for 1 h, followed by incubation with primary antibodies against ZO-1 (1:1000, Abcam, UK), occludin (1:50,000, Abcam, UK) and β-actin (1:1000, Santa Cruz, USA) overnight at 4 °C. After washing with PBST, the membranes were incubated with HRP-conjugated secondary antibodies (1:2000, Santa Cruz, USA) for 1 h at room temperature. After washing with PBST, immunoreactive bands were visualized using an ECL chemiluminescent kit (ECL Plus; Thermo Scientific, USA) and measured using Quality One analysis software (Bio-Rad, Hercules, USA). The density of each band was analyzed with ImageJ software. The relative expression of target proteins was normalized to β-actin.

### Gut microbiota analysis

Genomic DNA was extracted from the fecal samples using an E.Z.N.A. stool DNA Kit (Omega Biotek, Norcross, GA, USA) according to the manufacturer’s protocols. The 16S rRNA V3–V4 region of the eukaryotic ribosomal RNA gene was amplified by PCR. The primer sequences were as follows: 341F: CCTACGGGNGGCWGCAG; 806R: GGACTACHVGGGTATCTAAT. The PCR conditions used were an initial step at 95 °C for 2 min; followed by 27 cycles at 98 °C for 10 s, 62 °C for 30 s, and 68 °C for 30 s; and a final extension at 68 °C for 10 min. Amplicons were purified and then sequenced on the Hiseq2500 PE250 platform. Sequences were analyzed using Quantitative Insights into Microbial Ecology (QIIME)^20^. High-quality reads were selected, and all of the effective reads were clustered into operational taxonomic units (OTUs). Sequences with ≥97% similarity were assigned to the same OTUs by the Ribosomal Database Project (RDP) classifier 2.2 based on the SILVA Database^21^. Alpha diversity analysis (Chao1 and Shannon indices) was calculated using QIIME as described previously^20^. Beta diversity was calculated from unweighted UniFrac distances. Hierarchical clustering of samples was completed by using the unweighted pair-group method with arithmetic mean (UPGMA). Principal coordinate analysis (PCoA) on unweighted UniFrac distance matrices was performed to display the differences in composition and structure of the gut microbiota using R software. To identify different taxon microbes in the groups, linear discriminate analysis effect size (LEfSe) analyses were performed. Phylogenetic Investigation of Communities by Reconstruction of Unobserved States (PICRUSt) was used to predict the functional profiles of the microbial communities on the basis of the data in the Kyoto Encyclopedia of Genes and Genomes (KEGG) pathway database ^22^.

### Metabolomics analysis

The serum sample (100 μL) was added to 300 μL of prechilled methanol (50%), and the mixture was vortexed for 15 s and centrifuged at 12,000 rpm for 15 min at 4 °C. The supernatant was transferred and evaporated to dryness under nitrogen. The residue was dissolved in 100 μL of prechilled methanol. After centrifugation again at 12,000 rpm for 15 min at 4 °C, the supernatant was transferred to a sample vial for UPLC/Q-TOF-MS analysis. The detailed methods are provided in the Supplementary Information.

### Short-chain fatty acid (SCFA) analysis

SCFAs (acetic acid, propionic acid, butyric acid, isobutyric acid, valeric acid, and isovaleric acid) in the feces were analyzed by GC as described previously^23^. In brief, fecal samples (300 mg) were homogenized in 1.5 mL of deionized water and centrifuged at 12,000 × *g* for 5 min at 4 °C. A mixture of the supernatant and formic acid (300 and 30 μL, respectively) was then filtered with a 0.45-μm polysulfone filter membrane for assay. In addition, standard solutions of SCFAs (Sigma-Aldrich, USA) at different concentrations were prepared in deionized water. All assays were performed on an Agilent 7820A gas chromatograph (Agilent Technologies, Inc., USA) with a flame ionization detector (FID). The separation was achieved using a DB-FFAP 122-3232 column, 30 m × 0.25 mm × 0.25 µm (Agilent Technologies Inc., USA). The initial oven temperature was 50 °C and then raised to 120 °C at a rate of 15 °C/min and then further increased to 170 °C at a rate of 5 °C/min. The temperature was further increased to 210 °C at a rate of 15 °C/min and remained for 3 min. The temperature of the injection port and FID were set at 240 °C and 200 °C, respectively. Helium was used as the carrier gas at a flow rate of 1 mL/min. The injected sample volume was 1 μL. The standard curves of the target SCFAs were designed to quantify the SCFAs in the feces.

### Statistical analysis

Data are expressed as mean ± SD. Statistical analyses were performed using SPSS 16.0 software. Statistical differences were analyzed by one‐way ANOVA followed by the Tukey test. Pearson’s correlation was used to show the relations between parameters. *P* < 0.05 was considered statistically significant.

## Results

### NUC prevents HFD-induced obesity in rats

To investigate the effects of NUC on obesity, we established an obese rat model by feeding rats a HFD for 8 weeks. HFD feeding for this period led to significant increases in body and liver weight, epididymal and perirenal fat accumulation, and lipid deposition in adipocytes compared with the ND group (Fig. 1b–f, i, j). Notably, NUC supplementation significantly decreased body weight and fat accumulation in HFD-fed rats (Fig. 1b–f, i, j). HFD-fed rats provided NUC for 8 weeks showed a significant reduction in body and liver weight compared to unsupplemented HFD rats (Fig. 1b, d). In addition, NUC treatments significantly decreased both epididymal and perirenal fat weight and epididymal fat area in HFD-fed rats compared with the HFD group (Fig. 1e, f, i, j). Mean food intake showed no statistically significant differences between the HFD groups (Fig. 1g, h), suggesting that the effects of NUC on body weight and fat accumulation were not due to reduced food consumption. The HFD + SIM group showed the same pattern for the above parameters associated with obesity as the HFD + NUC group. There was no significant change in weight gain or fat accumulation between the ND and ND + NUC groups (Supplementary Information Fig. S1). Altogether, these results indicate that NUC reduces weight gain and fat accumulation in HFD-fed rats.

### NUC ameliorates lipid metabolic disorder in HFD-fed rats

**Fig. 2:**
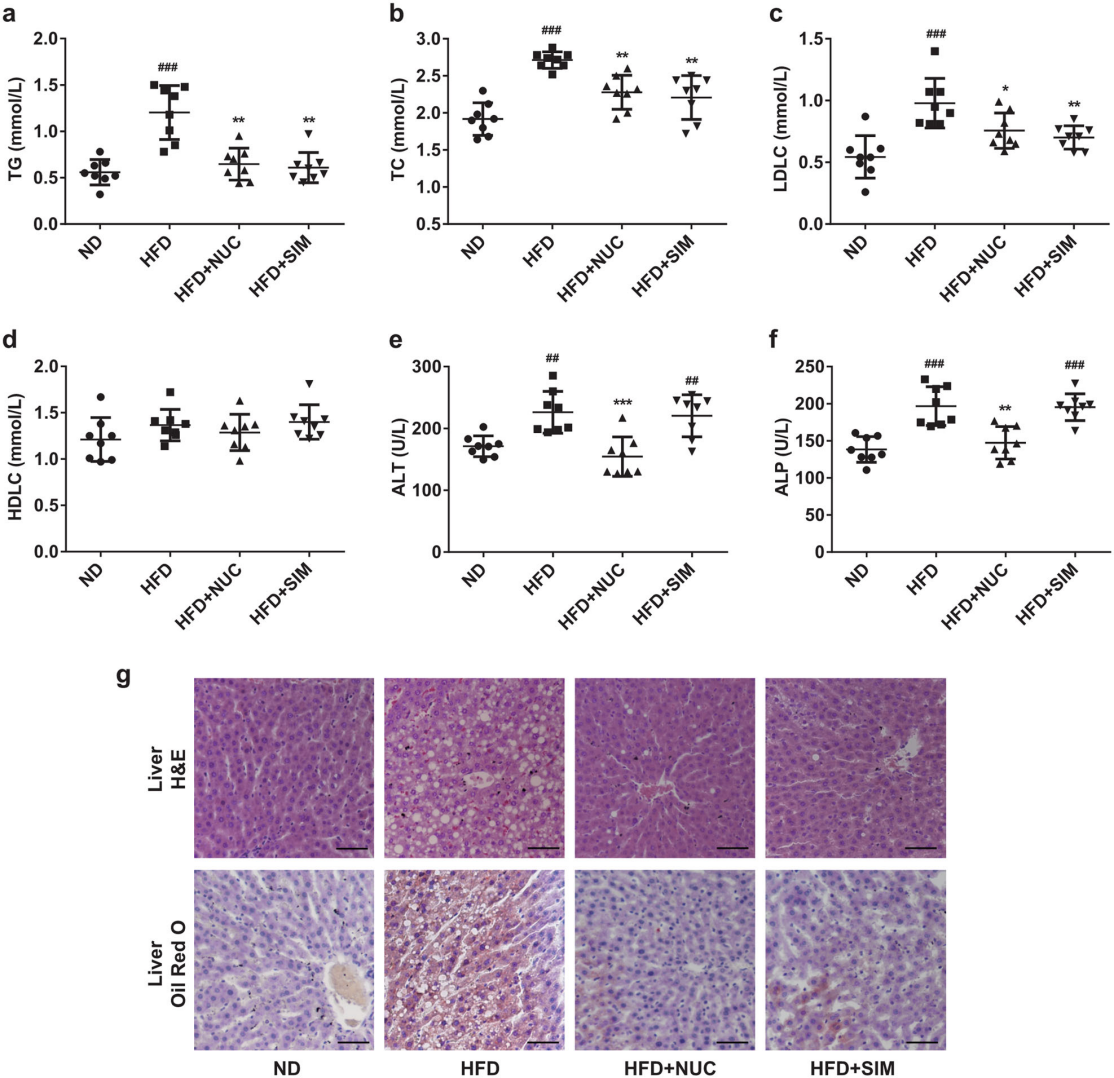
NUC improves the lipid profile and liver function and prevents hepatic fatty deposition in HFD-fed rats. Serum levels of TG (**a**), TC (**b**), LDL-C (**c**), HDL-C (**d**), ALT (**e**), and ALP (**f**) were determined. Liver lipid content (**g**) was assessed using H&E staining (magnification = 200×, scale bar, 50 μm) and Oil Red O staining (magnification = 200×, scale bar, 50 μm). Values are presented as the mean ± SD (*n* = 8 per group). ^##^*P* < 0.01, ^###^*P* < 0.001 vs ND, *P < 0.05, **P < 0.01, ***P < 0.001 vs HFD.

Relevant lipid metabolism and liver function parameters were detected in the four experimental groups. After 8 weeks, NUC treatment significantly decreased serum TG, TC, and LDL-C levels in comparison with the HFD group (Fig. 2a–c). In particular, the TG level in HFD-fed rats with NUC was decreased by 46% in unsupplemented HFD-fed rats (Fig. 2a). Furthermore, there were no obvious differences in the serum levels of HDL-C among groups (Fig. 2d). The HFD + SIM group showed the same pattern for these biochemical indices as the HFD + NUC group. After NUC treatment, the serum ALT and ALP levels were significantly decreased (Fig. 2e, f), indicating that NUC improved liver function. However, there was no significant change in the liver function parameters between the HFD and HFD + SIM groups. In addition, the amounts of lipid droplets in the liver were reduced by H&E staining and Oil Red O staining after NUC treatment, implicating that HFD-induced hepatic fat accumulation could be reduced by NUC (Fig. 2g). NUC had no significant effects on lipid metabolism and liver function in ND-fed rats (Supplementary Information Fig. S2). Thus, these results demonstrate that NUC improves the lipid profile, hepatic steatosis and liver injury in HFD-fed rats.

### NUC alters the diversity and composition of gut microbiota in HFD-fed rats

#### Analysis of the diversity of the gut microbiota

**Fig. 3:**
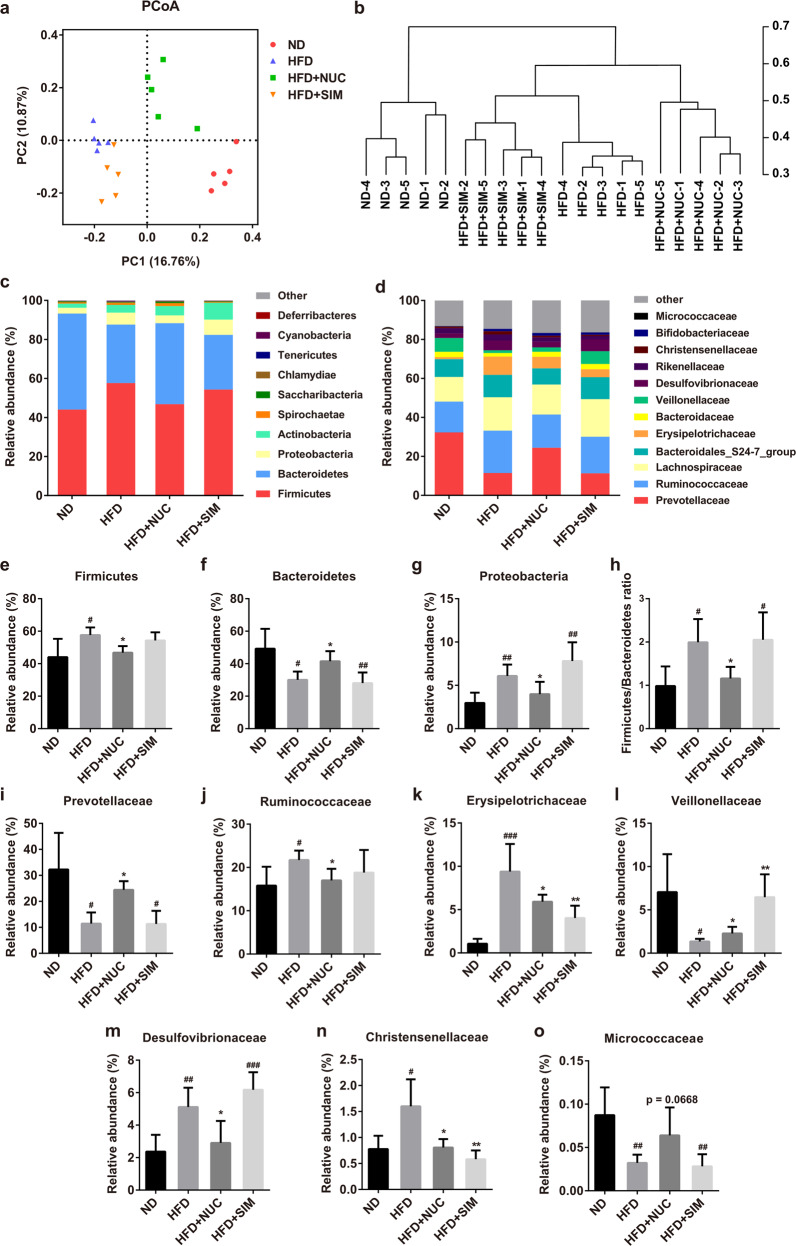
NUC alters the diversity and composition of gut microbiota in HFD-fed rats. **a** Unweighted UniFrac PCoA plot based on the OTU abundance of each rat. **b** UPGMA analysis based on (**a**). Bacterial taxonomic profiling at the phylum (**c**) and family (**d**) levels of intestinal bacteria from different groups. The relative abundance of the bacterial phylum (**e**–**h**) and family (**i**–**o**) changes in the fecal samples from different groups. Values are presented as the mean ± SD (*n* = 5 per group). ^#^*P* < 0.05, ^##^*P* < 0.01, ^###^*P* < 0.001 vs ND, *P < 0.05, **P < 0.01 vs HFD.

Accumulating evidence has suggested that gut microbiota might be a potential target for the treatment of obesity and related metabolic diseases^5,6^. We examined whether NUC may alter the composition and diversity of the gut microbiota by analyzing bacterial 16S rRNA (V3–V4 region) in feces. Analysis of the α-diversity indices (Chao1 and Shannon) reflecting species richness and diversity indicated that the sequencing depth covered rare new phylotypes and most of the diversity (Supplementary Information Fig. S3). There were no significant differences in species abundance and diversity among the various groups. β-Diversity represents compositional differences between samples (Fig. 3a, b). PCoA of unweighted UniFrac distances performed on the OTU abundance matrix showed that the β-diversity of gut microbial communities was significantly different among the three groups (ND, HFD, and HFD + NUC), and microbial composition was significantly influenced by both HFD and NUC supplementation (Fig. 3a). UPGMA analysis indicated that NUC supplementation shifted the overall structure of the gut microbiota of HFD-fed rats toward that of ND-fed rats (Fig. 3b). Interestingly, there was no significant change in the gut microbiota structures between the HFD and HFD + SIM groups, even though body weight and fat accumulation were significantly reduced and the lipid profile and hepatic fatty deposition were improved in the HFD + SIM group. This finding indicated that the anti-obesity effect of simvastatin was not caused by modulating the gut microbiota.

#### Analysis of the whole gut microbiota composition

Firmicutes and Bacteroidetes were the two main phyla in each group. The ratio of Firmicutes and Bacteroidetes was highly associated with obesity^7^. Proteobacteria (members of which are commonly found in the human gut microbiota), such as the families *Enterobacteriaceae* and *Desulfovibrionaceae*, are considered the main pathogenic bacteria producing endotoxins^24^. The increased concentration of these LPS-producing bacteria is believed to be the reason for the increased level of endotoxins and liver damage observed in HFD-fed mice^25^. The distribution of bacterial taxa and the relative abundance of bacteria at the phylum level are shown in Fig. 3c. The taxonomic abundance indicated a significant increase in Firmicutes and Proteobacteria and a decrease in Bacteroidetes in HFD-fed rats compared with ND-fed rats (Fig. 3e-g). However, NUC supplementation restored these levels and significantly reduced the ratio of Firmicutes to Bacteroidetes in HFD-fed rats to levels similar to those of ND-fed rats (Fig. 3h). However, there was no significant change in the phylum level of gut microbiota between the HFD and HFD + SIM groups.

**Fig. 4:**
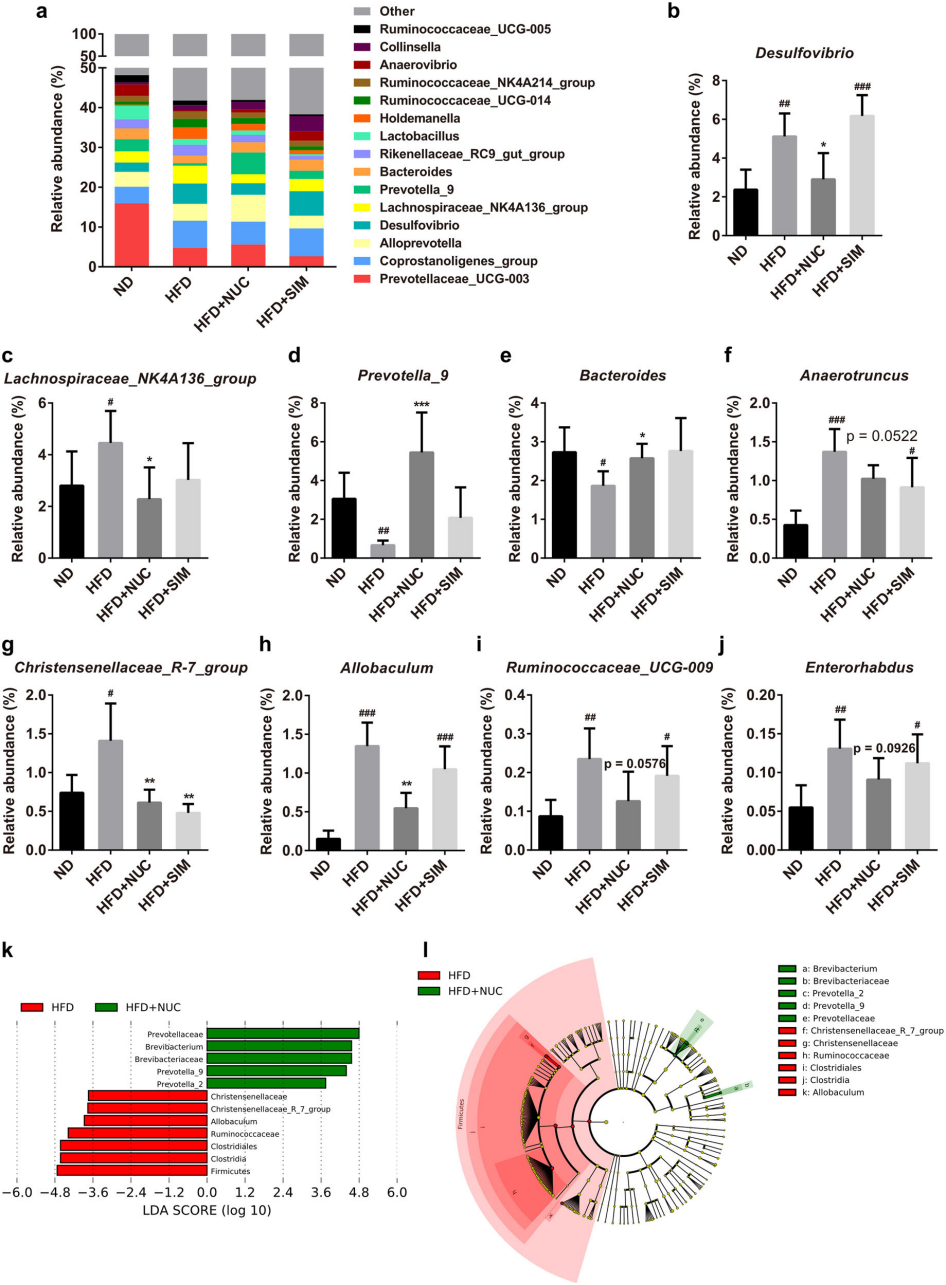
Effects of oral NUC on the abundance of gut microbiota in HFD-fed rats. Bacterial taxonomic profiling at the genus level of intestinal bacteria from different groups (**a**) is shown. The relative abundances of *Desulfovibrio* (**b**), *Lachnospiraceae_NK4A136_group* (**c**), *Prevotella_9* (**d**), *Bacteroides* (**e**), *Anaerotruncus* (**f**), *Christensenellaceae_R-7_group* (**g**), *Allobaculum* (**h**), *Ruminococcaceae_UCG-009* (**i**), and *Enterorhabdus* (**j**) are shown. Linear discriminant analysis (LDA) scores (**k**) and the cladogram (**l**) were generated from linear discriminate analysis effect size (LEfSe) analysis, showing the biomarker taxa (LDA score of >2 and a significance of *P* < 0.05 determined by the Wilcoxon signed-rank test). In (**l**), the species with no significant difference were uniformly colored yellow, and the red node and green node represent the bacteria that played an important role in the red group and green group, respectively. Values are presented as the mean±SD (*n* = 5 per group). ^#^*P* < 0.05, ^##^*P* < 0.01, ^###^*P* < 0.001 vs ND, *P < 0.05, **P < 0.01, ^***^P < 0.001 vs HFD.

At the family level, Fig. 3d presents the taxonomic distributions of the microbial communities. NUC supplementation significantly decreased the abundance of *Ruminococcaceae*, *Erysipelotrichaceae*, *Desulfovibrionaceae*, and *Christensenellaceae* and significantly increased the abundance of *Prevotellaceae* and *Veillonellaceae* in HFD + NUC rats compared to HFD-fed rats (Fig. 3i–n). Simvastatin significantly altered the abundance of *Erysipelotrichaceae*, *Veillonellaceae* and *Christensenellaceae* in HFD + SIM rats (Fig. 4k, l, n). Moreover, NUC supplementation changed the level of Micrococcaceae in HFD + NUC rats, although it did not reach a significant level (Fig. 3o).

At the genus level, highly abundant species were selected, and the expression profiles of each group were analyzed (Fig. 4a). Fifty genera with relative abundances are presented in a heat map (Supplementary Information Fig. S4). Apparently, genera showed different abundances among the four groups. The heat map of the relative abundances of microbial species altered by NUC treatment shows the differences in the gut bacterial compositions compared to the HFD group at the genus level. NUC supplementation for 8 weeks significantly reduced the levels of *Desulfovibrio*, *Lachnospiraceae_NK4A136_group*, *Christensenellaceae_R-7_group*, and *Allobaculum* compared with the HFD group (Fig. 4b, c, g, h). NUC supplementation also reduced the levels of *Anaerotruncus*, *Ruminococcaceae_UCG-009,* and *Enterorhabdus* in HFD + NUC rats, although the difference was not significant (Fig. 4f, i, j). The taxonomic abundance indicated a significant decrease in *Prevotella_9* and *Bacteroides* in HFD vs. ND rats, while NUC supplementation altered the genus levels in HFD + NUC *vs*. HFD rats (Fig. 4d, e). Simvastatin significantly decreased the abundance of *Christensenellaceae_R-7_group* in HFD + SIM rats (Fig. 4g). In addition, the linear discriminant analysis (LDA) effect size (LEfSe) method was used to identify statistically significant biomarkers and dominant microbiota between the HFD group and HFD + NUC group. Notably, Firmicutes was found to be the major phylum of gut microbiota in the HFD group, while *Prevotellaceae* and *Prevotella_9* were found in the HFD + NUC group (Fig. 4k, l). There was no significant difference in the diversity and composition of the gut microbiota between the ND and ND + NUC groups (Supplementary Information Figs. S5–6).

It has been reported that a bloom of *Erysipelotrichaceae*^26^, *Ruminococcaceae*^27^, *Enterorhabdus* (*Coriobacteriaceae*)^28,29^, *Lachnospiraceae_NK4A136_group*, and *Anaerotrucus*^30,31^, which were involved in host lipid metabolism, was described in diet-induced obese animals and individuals. Healthy gut levels of *Prevotella* and *Bacteroides* are well known to produce SCFAs^32,33^. Collectively, these results showed that NUC alters the gut microbiota in HFD-fed rats and mainly prevents HFD-induced elevation of LPS-producing bacteria and associated lipid metabolism and the reduction of SCFA-producing bacteria.

#### Prediction of potential metabolic functions of gut microbiota and the effects of NUC on lipid metabolism gene expression and serum metabolites in HFD-fed rats

**Fig. 5:**
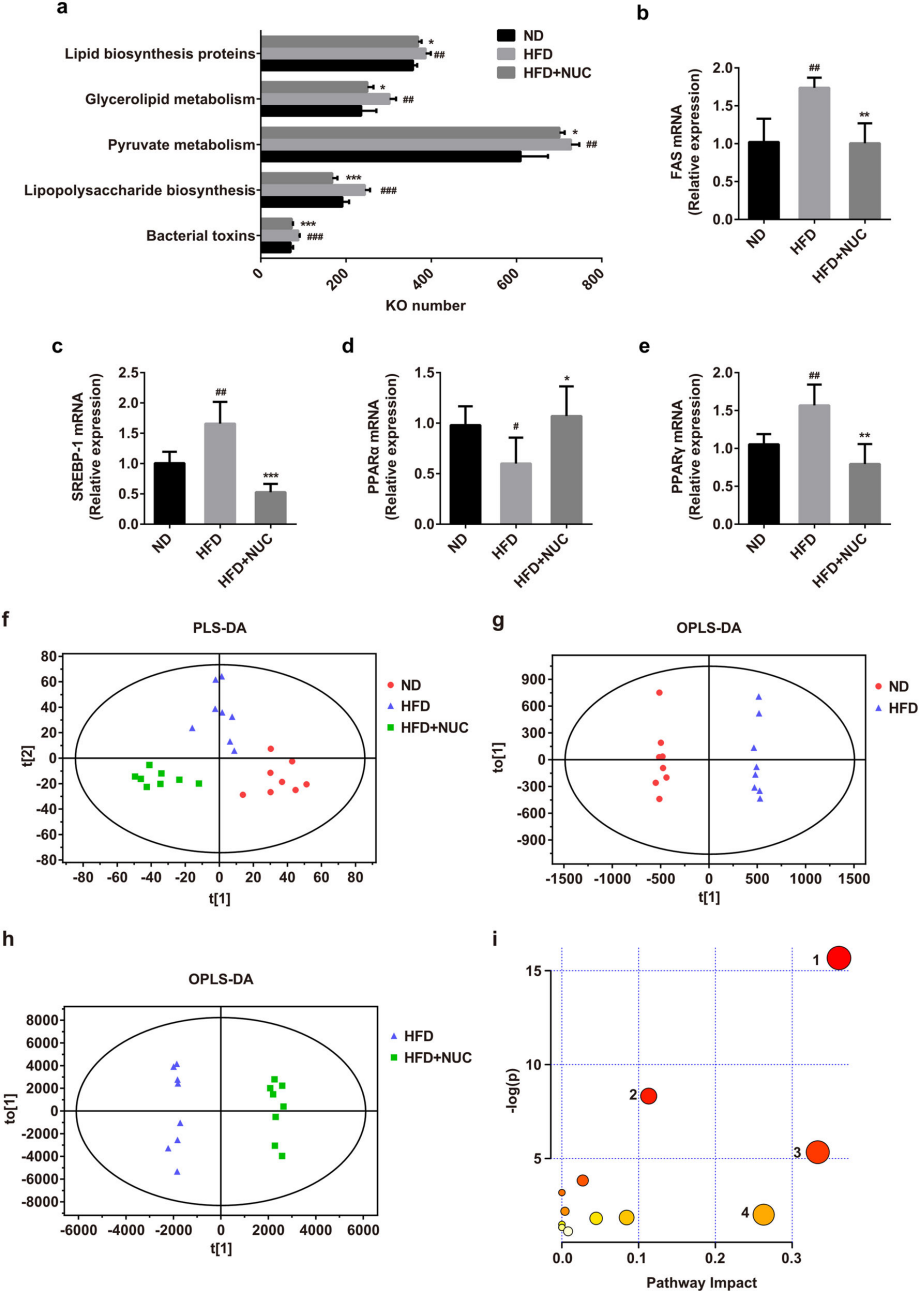
Prediction of potential metabolic functions of gut microbiota and the effects of NUC on lipid metabolism gene expression and serum metabolites in HFD-fed rats. Changes in the effect of NUC supplementation on the functional potential of the gut microbiome in HFD-fed rats (**a**) are shown. Metabolic pathways from KEGG module predictions using 16S rRNA data with PICRUSt (*n* = 5 per group). The effects of NUC treatment on the mRNA expression of FAS (**b**), SREBP-1 (**c**), and PPARɑ/γ (**d** and **e**) were monitored in the liver using qRT-PCR (*n* = 5 for each group) in comparison with the ND group. Expression was normalized against GAPDH. PLS-DA score plot of serum samples collected from different treatment groups of rats in positive ion mode (**f**), OPLS-DA score plot of the ND group vs HFD group (**g**) and the HFD group *vs* HFD + NUC group (**h**) in positive ion mode. **i** Summary of pathway analysis with MetaboAnalyst, including glycerophospholipid metabolism (1), glycerolipid metabolism (2), linoleic acid metabolism (3), and retinol metabolism (4) from significantly differential metabolites. The size and color of each circle are based on the pathway impact value and *P* value, respectively. Values are presented as the mean ± SD (*n* = 5 per group). ^#^*P* < 0.05, ^##^*P* < 0.01, ^###^*P* < 0.001 vs ND, *P < 0.05, **P < 0.01, ***P < 0.001 vs HFD.

Predicted functional metagenomic profiles based on KEGG pathways were generated using PICRUSt. Comparison between groups revealed significant differences in 40 predicted metabolic functions (Supplementary Information Fig. S7). Most of these features revealed similar abundances in ND rats compared with HFD + NUC rats. The correlations between bacterial abundance and predicted metagenomic function indicate that lipid biosynthesis proteins, glycerolipid metabolism, pyruvate metabolism, lipopolysaccharide biosynthesis, and bacterial toxins were modified in HFD + NUC rats compared with HFD rats (Fig. 5a). Thus, NUC alters potential metabolic functions of gut microbiota mainly involving lipid and carbohydrate metabolism and glycan biosynthesis and metabolism.

To further explore whether lipid metabolism was changed in association with changes in the metabolic pathways of microbial communities, we measured the mRNA expression levels of several genes related to lipid metabolism in the liver. Supplementation with NUC significantly reduced the expression levels of FAS, SREBP-1, and PPARγ and significantly increased the PPARα expression level compared with those of the HFD group (Fig. 5b–e). Based on these results, NUC may reduce lipid accumulation by regulating the expression of genes involved in lipid metabolism in HFD-fed rats.

Metabolomics has been shown to be a good tool to understand host–gut microbiota relationships^34^. Therefore, the metabolic profiles of serum samples from ND-fed rats and HFD-induced obese rats with and without NUC treatment were characterized using UPLC/Q-TOF-MS in positive ion scan mode. The data obtained from serum samples were analyzed by partial least squares discriminant analysis (PLS-DA). As shown in Fig. 5f, the PLS-DA score plots exhibited a distinct clustering of metabolites in serum samples of the three groups. The orthogonal partial least squares discriminant analysis (OPLS-DA) method was employed to sharpen an already established separation between the ND and HFD groups and between the HFD and HFD + NUC groups in PLS-DA (Fig. 5g, h). The results indicated that both diet and NUC influenced metabolic profiles.

To investigate the metabolic involvement in the therapeutic effect of NUC, the key differential metabolites were determined based on the criteria of both variable importance in the projection (VIP) > 1 and *P* < 0.05 in Student’s *t* test between the ND and HFD groups and were also significantly restored by NUC treatment compared to the HFD group. We identified a total of 32 differential metabolites as having significant metabolic profiles between the ND and HFD groups, as shown in Supplementary Information Table S2. Twenty-three key differential metabolites in the HFD group were differentially altered in the HFD + NUC group, indicating that NUC effectively improved HFD-induced serum metabolism disorder. The decreased differential metabolites in the HFD + NUC group mainly belonged to lysophospholipids (LysoPCs) including LysoPC(18:1), LysoPC(22:1), LysoPC(22:5), and LysoPC(P-18:0); phospholipids (PCs) including PC(15:0/22:0), PC(16:0/16:0) and PC(18:1e/2:0); phosphatidylethanolamines (PEs) including PE(20:0/dm18:0) and PE(22:5/P-18:1); and lysobisphosphatidic acids (LPAs) including LPA(0:0/18:1) and LPA(0:0/18:2), as well as diglyceride (DG)(15:0/20:5/0:0), TG(20:5/18:3/20:5), glycocholic acid, undecaprenyl diphosphate, vitamin A, and sphingosine. In addition, 6 metabolites, including LysoPC (16:0), LysoPC (P-18:1), PE (18:1/24:1), PE (22:1/P-18:1), linolenic acid, and phosphatidylglycerophosphate (PGP) (16:0/22:5), were significantly increased in the HFD + NUC group. To explore the possible metabolic pathways influenced by NUC, metabolic pathway analysis was carried out on MetaboAnalyst. Pathway impact plots were built to visualize the impact of altered metabolic pathways (Fig. 5i). Pathways with an impact value >0.1 were considered potential target pathways. Accordingly, the major metabolic pathways in serum samples involved glycerophospholipid metabolism, glycerolipid metabolism, linoleic acid metabolism, and retinol metabolism. All of these altered metabolites and metabolic pathways suggested that NUC effectively improved HFD-induced disorder of endogenous metabolism, especially lipid metabolism.

### NUC promotes SCFA production, enhances intestinal barrier integrity and reduces inflammation in HFD-fed rats

**Fig. 6:**
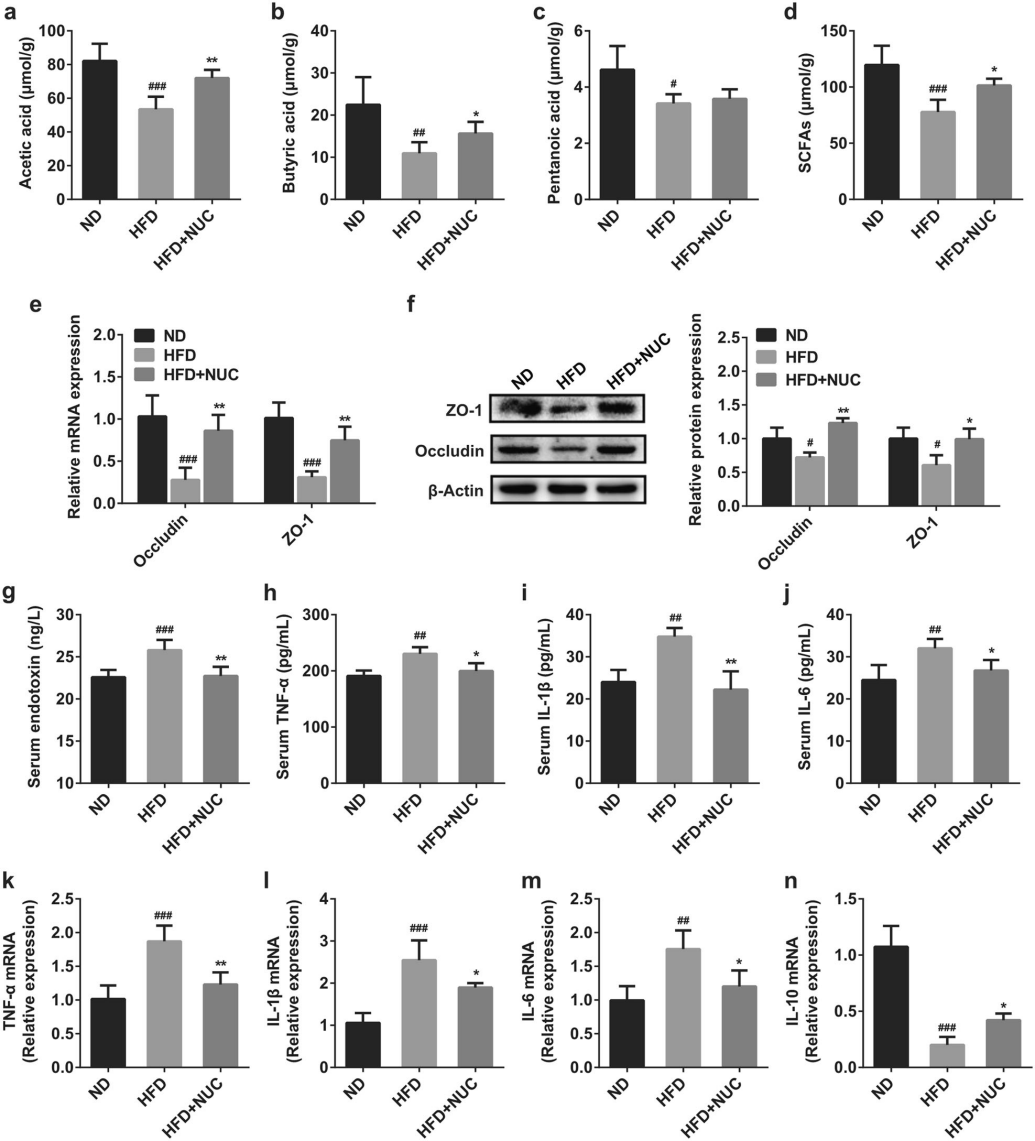
NUC increases SCFA production and intestinal tight junction expression and decreases serum LPS levels and proinflammatory cytokine expression and production in HFD-fed rats. Acetic acid (**a**), butyric acid (**b**), pentanoic acid (both n-pentanoic and isopentanoic acids) (**c**) and SCFA (**d**) levels in feces were determined by GC. The effects of NUC treatment on the mRNA expression levels of occludin and ZO-1 (**e**) as well as on the amount of the corresponding proteins (**f**) in the colon were determined. Relative mRNA expression in (**e**) was monitored using qRT-PCR in comparison with the ND group. Expression was normalized against GAPDH. Representative immunoblots for ZO-1, occludin and β-actin (**f**) in each group are shown. β-Actin levels were used as a loading control. Bar graphs show densitometry analysis of specific bands relative to the ND group (*n* = 3 per group). Serum LPS levels (**g**) were determined. Relative expression levels of TNF-α (**a**), IL-1β (**b**), IL-6 (**c**), and IL-10 (**d**) in hepatic tissue were assessed using qRT-PCR. TNF-α (**e**), IL-1β (**f**) and IL-6 (**g**) protein levels in the serum of ND-fed and HFD-fed rats were determined using ELISA. Values are presented as the mean ± SD (*n* = 5 per group). ^#^*P* < 0.05, ^##^*P* < 0.01, ^###^*P* < 0.001 vs ND, *P < 0.05, **P < 0.01 vs HFD.

Obesity is accompanied by a decrease in SCFAs (mainly acetate, propionate, butyrate, etc.) produced by the intestinal microbiota and damage to intestinal barrier integrity^35,36^. This process leads to the release of bacterial LPS into the circulation, in turn leading to obesity and obesity-related dysfunctions^8^. It has been reported that an increase in SCFA concentration improves intestinal barrier integrity^37^. As shown in Fig. 6a–d, NUC supplementation significantly increased the concentration of fecal SCFAs, especially acetic acid and butyric acid. Tight junction proteins are the main factors that contribute to intestinal integrity^38^. We therefore measured the expression of the major tight junction proteins ZO-1 and occludin in colon tissue. Although HFD feeding reduced the expression of ZO-1 and occludin in the colon tissue, these effects were reversed by NUC supplementation (Fig. 6e, f). Similarly, rats in the HFD + NUC group had dramatically lower serum levels of LPS than rats in the HFD group (Fig. 6g). These results indicate that NUC promotes SCFA production and enhances intestinal barrier integrity in HFD-fed rats.

Previous reports have shown that HFD-fed obese animals produced higher levels of proinflammatory cytokines and lower levels of anti-inflammatory cytokines in hepatic tissue compared with ND-fed animals^39^. Notably, NUC supplementation reduced TNF-α, IL-1β, and IL-6 expression levels and increased IL-10 expression levels in hepatic tissue of HFD-fed rats (Fig. 6k–n). Moreover, NUC supplementation reduced the levels of secreted TNF-α, IL-1β and IL-6 proteins in the serum of HFD-fed rats (Fig. 6h–j). These results indicate that NUC supplementation reduces inflammation markers in obese rats.

### Potential relations between disease biomarkers and gut microbiota

**Fig. 7:**
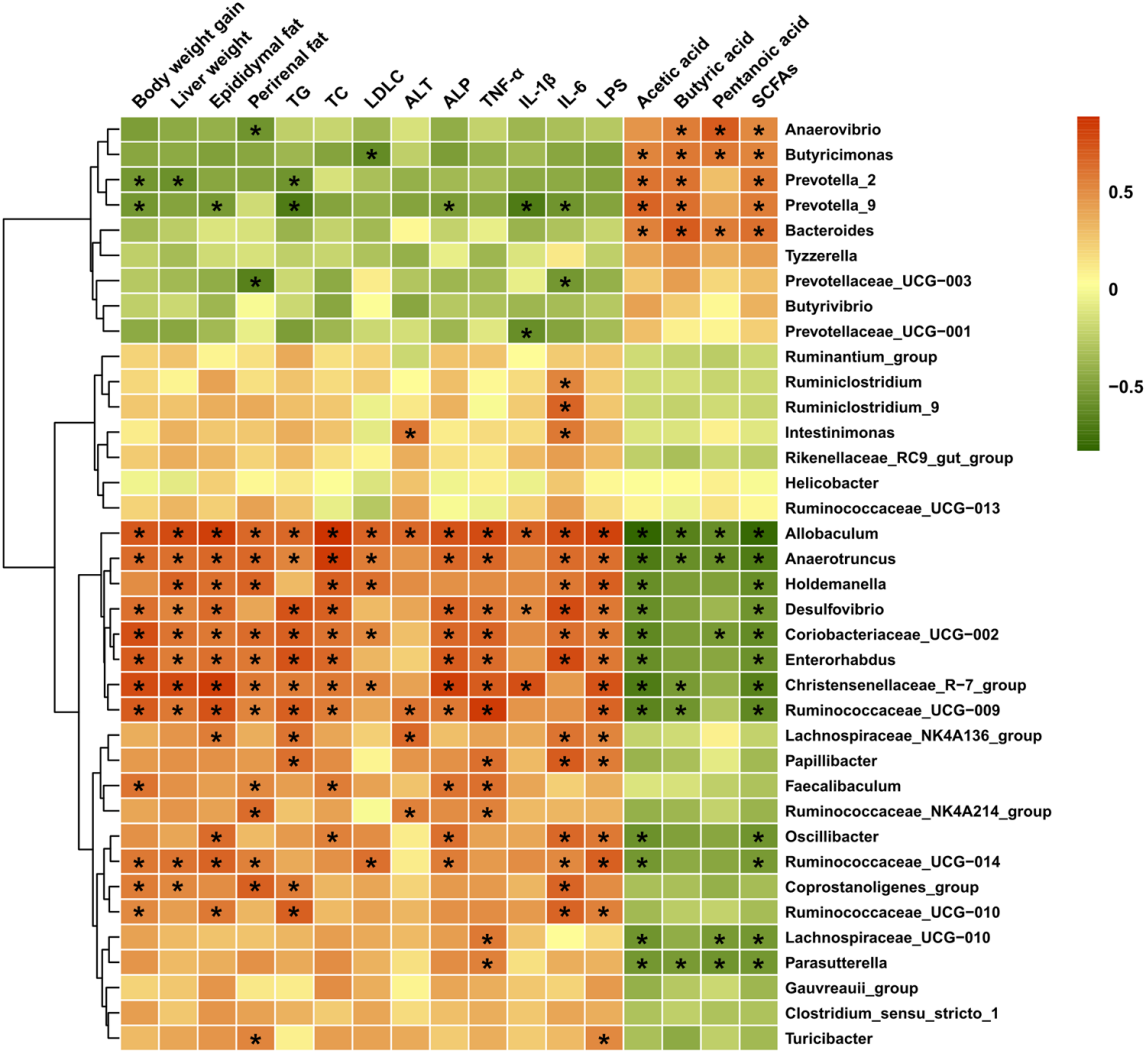
The correlation of gut microbiota changes with clinical parameters related to obesity was presented as a heat map analysis. Pearson correlation values were used for the matrix, with red indicating a positive correlation and green indicating a negative correlation. “*” Denotes adjusted *P* < 0.05.

To comprehensively analyze the relations between disease biomarkers and gut microbiota, a correlation matrix was generated by calculating Pearson’s correlation coefficient. As shown in Fig. 7, 29 of the 37 genera reversed by NUC intervention were significantly negatively or positively associated with at least one parameter of obesity. *Allobaculum*, *Anaerotruncus*, *Desulfovibrio*, *Coriobacteriaceae_UCG-002*, *Enterorhabdus*, *Christensenellaceae_R-7_group*, *Ruminococcaceae_UCG-009*, *Ruminococcaceae_UCG-014*, and *Coprostanoligenes_group* were significantly positively associated with body weight gain, liver weight, and epididymal or perirenal fat accumulation. *Allobaculum*, *Anaerotruncus*, *Coriobacteriaceae_UCG-002*, and *Christensenellaceae_R-7_group* were significantly positively correlated with serum TG, TC, and LDL-C levels. *Allobaculum*, *Anaerotruncus*, *Desulfovibrio*, *Coriobacteriaceae_UCG-002*, *Enterorhabdus*, and *Papillibacter* were significantly positively correlated with serum TNF-α, IL-6, and LPS levels. *Butyricimonas*, *Prevotella_2*, *Prevotella_9*, and *Bacteroides* were significantly positively correlated and *Allobaculum*, *Anaerotruncus*, *Christensenellaceae_R-7_group*, *Ruminococcaceae_UCG-009*, and *Parasutterella* were significantly negatively correlated with the fecal acetic and butyric acid and SCFA levels. These relations suggested that gut microbiota could affect not only host phenotypes but also the serum lipid profile, liver function parameters, inflammation markers, and SCFA levels. Taken together, the results demonstrated that NUC intervention could modulate HFD-induced gut microbiota dysbiosis, resulting in a healthy gut microbiota composition similar to that of the ND group.

## Discussion

Although previous reports have shown that NUC ameliorated hepatic steatosis in HFD-induced hamsters and HFD/streptozotocin-induced diabetic mice^16,18^, the effect of NUC on gut microbiota in obesity had not been investigated. Modulation of the gut microbiota composition arises as a promising tool to prevent the development of obesity and related metabolic disorders^5^. In the present study, we found that NUC supplementation can prevent dietary-induced obesity, improve lipid metabolic disorders and reduce inflammation, and the potential mechanisms could be due to modulating the composition and potential function of the gut microbiota, promoting SCFA production, and enhancing intestinal barrier integrity.

Experimental studies in animal models and in humans suggest that the gut microbiota is altered in obesity. In the present work, HFD feeding led to profound alterations in the diversity and composition of the gut microbiota based on the results of PCoA and hierarchical cluster analysis, which is in agreement with previous reports^40,41^. Analysis of the fecal microbiota demonstrated that NUC improved gut microbiota dysbiosis in HFD-fed rats. The gut microbiota of obese animals and patients is associated with increased levels of members of the Firmicutes phylum and decreased levels of the Bacteroidetes phylum, indicating that the two dominant bacterial phyla may play a role in obesity^7,8,42,43^. The increase in the Firmicutes/Bacteroidetes ratio in obese mice could be associated with a possible host-mediated adaptive response to limit energy uptake/storage and/or to promote adiposity^7^. However, NUC supplementation restored the relative abundances of Firmicutes and Bacteroides and the Firmicutes/Bacteroidetes ratio to that observed in ND-fed rats.

Martínez et al. observed that the levels of several bacterial taxa from *Erysipelotrichaceae* and *Coriobacteriaceae* displayed significantly high correlations with cholesterol metabolites in a hamster model^28,29^. Moreover, some *Coriobacteriaceae* are involved in bile acid metabolism, which is linked to gut barrier and metabolic dysfunctions^44,45^. In this study, the abundance of the genus *Coriobacteriaceae_UCG-002* was significantly positively correlated with serum TG, TC, and LDL-C levels, and the abundance of the family *Erysipelotrichaceae* and the genus *Enterorhabdus* within *Coriobacteriaceae* was decreased by treatment of HFD rats with NUC. Kim et al.^27^ observed that *Ruminococcaceae* was enriched in HFD mice. Interestingly, the abundance of the genus *Ruminococcaceae_UCG-009* was significantly positively correlated with the lipid profile in our study. The increased abundance of *Ruminococcaceae* as well as its genus *Ruminococcaceae_UCG-009* induced by HFD can be alleviated by NUC treatment, which may be related to the decrease in the lipid profile in the NUC group. Our research also suggested that the abundance of *Lachnospiraceae_NK4A136_group* and *Anaerotrucus* decreased after NUC supplementation in HFD rats, which have been reported to be associated with obesity and other associated metabolic disorders^30,31^. A colonization experiment demonstrated the positive role of *Lachnospiraceae* (a family of Clostridia) in the development of obesity and diabetes in germ-free *ob*/*ob* mice, including increased liver and adipose tissue weights and fasting blood glucose levels and decreased plasma insulin levels and homeostasis model assessment of β-cell function (HOMA-β) values^30^.

The predictive tool PICRUSt infers the metabolic activities of the microbiota by comparing the composition with reference genomes of known functional potential^22^. PICRUSt revealed significant differences in 40 predicted metabolic functions among the three groups. Furthermore, compared with HFD rats, HFD rats administered NUC exhibited increased functions mainly involving lipid and carbohydrate metabolism and glycan biosynthesis and metabolism. Here, we compared the mRNA expression of several lipogenesis- and lipolysis-related genes, including FAS, SREBP-1, and PPARɑ/γ, in the liver of rats that received ND, HFD, or HFD + NUC. NUC supplementation markedly reversed the alteration of the mRNA expression levels of these genes in rats fed a HFD. A body of evidence suggests that the gut microbiota affects calorie harvest and host fat storage^5,6^. The gut microbiota could inhibit the intestinal expression of fasting-induced adipose factor (FIAF) to promote TG deposition in adipocytes^46^. The gut microbiota increased hepatic lipogenesis with increased expression of acetyl-CoA carboxylase (ACC), FAS, carbohydrate response element-binding protein (ChREBP), and SREBP-1^47^. On the other hand, since functional interactions between the gut microbiota and host endogenous metabolism have been well demonstrated^48^, we further analyzed the serum metabolites. The analysis of serum metabolomics showed that the majority of the differential metabolites affected by NUC supplementation were involved in lipid metabolism, especially glycerophospholipid metabolism and glycerolipid metabolism, including PC, LysoPC, PE, LPA, DG, and TG. Similarly, NUC affected the glycerophospholipid, linoleic acid, alpha-linolenic acid, arginine and proline metabolism pathways as assessed by metabolomic analysis and regulated the gene expression of related key enzymes in the NAFLD rat model^17^. Abnormalities in lipid and fatty acid metabolism cause dyslipidemia, which is one of the main risk factors for metabolic diseases^49^. Accumulating evidence has shown that phospholipids play a vital role in glycolipid metabolism and in the development of metabolic diseases such as obesity, insulin resistance, type 2 diabetes mellitus and cardiovascular disease^50,51^. Based on the results of the metabolic pathway analysis and the predicted metabolic functions of the gut microbiota, we postulate that the improvement in lipid metabolism in the NUC-supplemented group was partly caused by the changes in the metabolic pathways of microbial communities.

Metabolic inflammation plays an essential role in the development of obesity and related metabolic diseases^52,53^. Moreover, overproduction of proinflammatory cytokines, such as TNF-α, IL-1β, and IL-6, might induce the development of chronic inflammation in obese animals^39^. However, NUC supplementation reduces inflammation markers in obese rats. Accumulating evidence has shown that gut microbiota dysbiosis is associated with obesity and related metabolic diseases^5,6^. In particular, the gut microbiota is believed to contribute to metabolic disorders via stimulation of chronic inflammation^54^. Endotoxin LPS is a major component of the outer cell membrane of Gram-negative bacteria. HFD-induced gut microbiota dysbiosis can alter gut permeability (leaky gut) and then increase the concentration of LPS in the blood, which causes low-grade inflammation and, ultimately, obesity and related metabolic diseases in rodents and humans^8,55^. Interestingly, we found that NUC intervention reduced the enrichment of genes involved in LPS biosynthesis and related proteins based on the predicted function by 16S rRNA sequencing and PICRUSt analysis. Here, a lower abundance of LPS-producing bacteria, including the phylum Proteobacteria, the family *Desulfovibrionaceae* and the genus *Desulfovibrio*, was discovered in the HFD + NUC group than in the HFD group, which may help alleviate inflammation. In addition, the modulation of gut microbiota after HFD strongly increased intestinal barrier permeability by reducing the expression of genes coding for tight junction proteins (ZO-1 and occludin)^8^. Our results showed that NUC supplementation significantly decreased the plasma levels of LPS and augmented the LPS-induced decreases in the expression of gut tight junction proteins. Therefore, NUC supplementation attenuated chronic inflammation in obese rats fed a HFD by simultaneously blocking the generation (decrease in Proteobacteria, *Desulfovibrionaceae*, and *Desulfovibrio*), trafficking (maintenance of intestinal barrier integrity), and pathophysiologic functions (decrease in proinflammatory cytokine expression and secretion) of LPS, resulting in an improvement in obesity.

Current evidence suggests that SCFAs, such as acetic acid, propionic acid, and butyric acid, which are derived from gut microbial fermentation of indigestible foods, have important metabolic functions and are crucial for intestinal health. These SCFAs are involved in the pathophysiology of obesity and related disorders by affecting the control of body weight via energy intake and energy harvesting, maintaining intestinal homeostasis, and linking with insulin sensitivity through the inflammatory response, lipid storage and adipose tissue function^10,56^. Moreover, SCFAs, as signaling molecules through G-protein coupled receptor 43 (GPR43) and GPR41 (GPRs), may prevent the body weight gain induced by HFD feeding^9^. Our results showed that NUC significantly increased the levels of fecal SCFAs in HFD-fed rats, especially those of acetic acid and butyric acid. In addition, at the genus level, we found that fecal SCFA concentrations were positively correlated with the abundance of *Butyricimonas* and *Prevotella_2*, and the abundance of *Prevotella_9* and *Bacteroides* was enhanced by treatment of HFD rats with NUC. Healthy gut levels of *Butyricimonas*, *Prevotella,* and *Bacteroides* are well known to produce SCFAs^32,33^. Acetate and propionate are known to stimulate adipogenesis in an adipocyte cell line and increase leptin release from adipose tissue in mice^57,58^. Butyrate could improve gut barrier function by modulating mucus production and the expression of proteins involved in tight junctions (ZO-1 and occludin)^59,60^. In addition, butyrate could inhibit the production of proinflammatory cytokines and activate regulatory T cells, leading to the alleviation of colitis^60^. Therefore, these results indicate that the beneficial effects of NUC against obesity may be largely due to increases in the populations of these SCFA-producing bacterial species and the gut barrier-enhancing effects of SCFAs. However, we have extensively illustrated the connection, but not the cause-effect relationship, between improved gut microbiota and the anti-obesity effects of NUC. Therefore, further studies of fecal transfer experiments are necessary to elucidate this association.

In summary, our results demonstrated that NUC supplementation could reduce HFD-induced obesity in rats, and the potential mechanisms could be due to alterations in the abundance of the most functionally relevant gut bacterial phylotypes that are associated with metabolic parameters. These findings provide important experimental evidence to develop NUC as a potential drug for preventing obesity and related metabolic disorders, and the gut microbiota may represent the target of the potential anti-obesity strategy of NUC.

## Supplementary information

Supplementary Information
