# Supplementary material for: Nuciferine modulates the gut microbiota and prevents obesity in high-fat diet-fed rats
Source: Exp Mol Med. 2020 Dec 1;52(12):1959–75. doi: 10.1038/s12276-020-00534-2 (PMC8080667; doi:10.1038/s12276-020-00534-2)
Supplement: Supplementary file 1 — Supplementary Information [file 12276_2020_534_MOESM1_ESM.docx]

**Supplementary Information**

***Nuciferine modulates the gut microbiota and prevents obesity in high-fat diet-fed rats***

**Supplementary information for materials and methods**

**Metabolomics Analysis**

Chromatographic separation was performed on a Waters ACQUITY UPLC H-Class system (Waters Corp., Milford, MA, USA) with an ACQUITY HSS T3 column (2.1mm × 100 mm, 1.8 μm) at a flow rate of 0.4 mL/min and 40 °C column temperature. The injection volume was 3 μL. The mobile phase comprised acetonitrile (Phase A) and water (Phase B) (containing 0.1% formic acid) and the optimized gradient elution program was set as follows: 0–2 min: 5%A; 2–3 min: 5-30%A; 3–15 min: 30-95%A; 15–17 min: 95%A. The eluent from the column was directed to the mass spectrometer with no splitting.

Mass spectrometry with an electrospray ionization source operating in positive ion mode was performed on a Waters Xevo G2 Q-TOF mass spectrometer (Waters Corp., Milford, MA, USA). The parameters were set as follows: capillary voltage, 2.5 kV; sample cone voltage, 45 V; desolvation gas rate and temperature, 800 L/h and 500 °C; cone gas rate, 50 L/h; scan time, 0.2 s. Leucine-enkephalin was used as the lock mass in all analyses ([M+H]^+^ = 556.2771) at a concentration of 0.2 μg/mL with a flow rate of 10 μL/min. Data was collected in centroid mode from m/z 50 to 1500. The quality control (QC) was prepared by mixing an equal amount of each sample to further monitor the system stability.

Progenesis QI V2.0 (Waters Corp., Milford, MA, USA) was used to analyze the raw data. After preprocessing (including integration, normalization and alignment the intensities of peaks), a list of the intensities of the peaks was generated on retention time and *m*/*z* data pairs obtained from the samples in the positive data set. The processed data list was then imported to EZinfo 3.0 software for orthogonal partial least squares discriminant analysis (OPLS-DA). Differential metabolites were selected based on a variable importance in the projection (VIP) value (VIP > 1) in the OPLS-DA model and *P*<0.05 in Student’s t test. The significantly changed metabolites (potential biomarkers), were interpreted using the Human Metabolome Database (HMDB) (http://www.hmdb.ca/), METLIN (https://metlin.scripps.edu/) and KEGG (http://www.genome.jp/kegg/pathway.html). MetaboAnalyst was also performed (http://www.metaboanalyst.ca/) to identify the affected metabolic pathways and facilitate further biological interpretation.

**Supplemental tables**

**Table S1.** The primer sequences used for real-time PCR analysis in our work.

| Gene | Forward primer (5′-3′) | Reverse primer (5′-3′) |
| --- | --- | --- |
| GAPDH | GGATCTCGCTCCTGGAAGATG | TACCAGGGCTGCCTTCTCTTG |
| TNF-α | ATGGGCTCCCTCTCATCAGT | TCCCTCAGGGGTGTCCTTAG |
| IL-1β | CAGCTTTCGACAGTGAGGAGA | TTGTCGAGATGCTGCTGTGA |
| IL-6 | TTCTCTCCGCAAGAGACTTCC | ACAGTGCATCATCGCTGTTC |
| IL-10 | TGCGACGCTGTCATCGATTT | GTAGATGCCGGGTGGTTCAA |
| Occludin | CCCTTCTTTCCTTAGGCGACC | TGGGTTTGAATTCATCCGGC |
| ZO-1 | GCCACACTGTGACCCTAAAAC | AGGACAGAAACACAGTTGGCT |
| FAS | CCCGGACCCAGAATACCAAG | TCTTCAAGTCCACACGAGGTG |
| SREBP-1 | CCATGGACGAGCTACCCTTC | AGCATGTCTTCGATGTCGGT |
| PPARα | GTCCTGGAACTGAAGCGACG | GCACCAATCTGTGATGACAACG |
| PPARγ | GGGAGATCCTCCTGTTGACC | ATCGCACTTTGGTATTCTTGGAG |

**Table S2.** Endogenous metabolite variations in serum induced by NUC supplementation in HFD induced obese rats.

| **No.** | **RT** | ***m*/*z*** | **Metabolite** | **Formula** | **HMDB ID** | **FC**^#^ | | **Trend** | |
| --- | --- | --- | --- | --- | --- | --- | --- | --- | --- |
|  |  |  |  |  |  | **HFD *vs* ND** | **HFD+NUC *vs* HFD** | **HFD *vs* ND** | **HFD+NUC *vs* HFD** |
| 1 | 9.42 | 301.2163 | Linolenic acid | C_18_H_30_O_2_ | HMDB0001388 | 0.57 | 1.74 | ↓* | ↑* |
| 2 | 12.82 | 303.2320 | Linoleic acid | C_18_H_32_O_2_ | HMDB0000673 | 0.49 | 1.13 | ↓* | ↑ |
| 3 | 11.94 | 623.4675 | DG(15:0/20:5/0:0) | C_38_H_64_O_5_ | HMDB0007085 | 1.99 | 0.50 | ↑* | ↓* |
| 4 | 6.89 | 465.3097 | Glycocholic acid | C_26_H_43_NO_6_ | HMDB0000138 | 4.43 | 0.40 | ↑*** | ↓*** |
| 5 | 13.32 | 436.2599 | LPA(0:0/18:1) | C_21_H_41_O_7_P | HMDB0007851 | 2.02 | 0.38 | ↑*** | ↓*** |
| 6 | 9.06 | 457.2353 | LPA(0:0/18:2) | C_21_H_39_O_7_P | HMDB0007852 | 3.08 | 0.44 | ↑** | ↓** |
| 7 | 10.64 | 495.3329 | LysoPC(16:0) | C_24_H_50_NO_7_P | HMDB0010382 | 0.47 | 1.60 | ↓** | ↑* |
| 8 | 11.12 | 522.3547 | LysoPC(18:1) | C_26_H_52_NO_7_P | HMDB0002815 | 2.51 | 0.54 | ↑** | ↓* |
| 9 | 11.13 | 546.3549 | LysoPC(20:3) | C_28_H_52_NO_7_P | HMDB0010393 | 2.11 | 0.91 | ↑* | ↓ |
| 10 | 14.71 | 577.4127 | LysoPC(22:1) | C_30_H_60_NO_7_P | HMDB0010399 | 1.75 | 0.51 | ↑* | ↓* |
| 11 | 10.85 | 569.3483 | LysoPC(22:5) | C_30_H_52_NO_7_P | HMDB0010402 | 2.19 | 0.63 | ↑* | ↓* |
| 12 | 11.93 | 608.4670 | LysoPC(24:0) | C_32_H_66_NO_7_P | HMDB0010405 | 1.99 | 0.94 | ↑* | ↓ |
| 13 | 13.15 | 508.3761 | LysoPC(P-18:0) | C_26_H_54_NO_6_P | HMDB0013122 | 3.11 | 0.48 | ↑** | ↓** |
| 14 | 10.71 | 506.3601 | LysoPC(P-18:1) | C_26_H_52_NO_6_P | HMDB0010408 | 0.39 | 2.80 | ↓** | ↑*** |
| 15 | 17.46 | 842.5977 | PC(15:0/22:0) | C_45_H_90_NO_8_P | HMDB0007953 | 2.95 | 0.40 | ↑** | ↓** |
| 16 | 17.36 | 756.5580 | PC(16:0/16:0) | C_40_H_80_NO_8_P | HMDB0000564 | 3.40 | 0.43 | ↑** | ↓* |
| 17 | 11.65 | 572.3711 | PC(18:1e/2:0) | C_28_H_56_NO_7_P | HMDB0011148 | 2.67 | 0.39 | ↑*** | ↓*** |
| 18 | 17.05 | 866.5973 | PE(18:1/24:1) | C_47_H_90_NO_8_P | HMDB0009047 | 0.42 | 1.70 | ↓** | ↑* |
| 19 | 18.02 | 798.5705 | PE(20:0/dm18:0) | C_43_H_86_NO_7_P | HMDB0009247 | 2.13 | 0.62 | ↑** | ↓** |
| 20 | 17.57 | 822.5743 | PE(22:1/P-18:1) | C_45_H_86_NO_7_P | HMDB0009545 | 0.47 | 2.01 | ↓** | ↑* |
| 21 | 12.30 | 814.5136 | PE(22:5/P-18:1) | C_45_H_78_NO_7_P | HMDB0009644 | 2.54 | 0.42 | ↑** | ↓*** |
| 22 | 6.27 | 915.4578 | PGP(16:0/22:5) | C_44_H_78_O_13_P_2_ | HMDB0013484 | 0.29 | 2.35 | ↓*** | ↑** |
| 23 | 4.10 | 837.4098 | PGP(16:1/16:1) | C_38_H_72_O_13_P_2_ | HMDB0013488 | 0.26 | 1.09 | ↓** | ↑ |
| 24 | 14.00 | 285.2220 | Retinal | C_20_H_28_O | HMDB0001358 | 0.36 | 1.15 | ↓** | ↑ |
| 25 | 9.61 | 300.2897 | Sphingosine | C_18_H_37_NO_2_ | HMDB0000252 | 2.05 | 0.39 | ↑** | ↓*** |
| 26 | 5.97 | 999.7696 | TG(14:0/24:1/22:6) | C_63_H_108_O_6_ | HMDB0042509 | 3.41 | 0.89 | ↑** | ↓ |
| 27 | 11.94 | 907.6826 | TG(15:0/18:4/22:6) | C_58_H_92_O_6_ | HMDB0043683 | 2.13 | 0.87 | ↑* | ↓ |
| 28 | 11.35 | 959.6484 | TG(20:5/18:3/20:5) | C_61_H_92_O_6_ | HMDB0010539 | 3.09 | 0.32 | ↑** | ↓** |
| 29 | 10.90 | 949.6290 | Undecaprenyl diphosphate | C_55_H_92_O_7_P_2_ | HMDB0001469 | 2.53 | 0.62 | ↑** | ↓** |
| 30 | 11.08 | 277.2162 | Palmitoleic acid | C_16_H_30_O_2_ | HMDB0003229 | 2.80 | 0.92 | ↑* | ↓ |
| 31 | 6.89 | 337.2513 | Nonadecanoic acid | C_19_H_38_O_2_ | HMDB0000772 | 2.72 | 0.90 | ↑** | ↓ |
| 32 | 11.82 | 286.2294 | Vitamin A | C_20_H_30_O | HMDB0000305 | 2.16 | 0.49 | ↑** | ↓*** |

^#^Fold change equals the fold difference in concentration observed between two groups.

↑: upregulated. ↓: downregulated. **P* < 0.05, ***P* < 0.01, ****P* < 0.001 *vs* HFD.

**Supplemental figures**


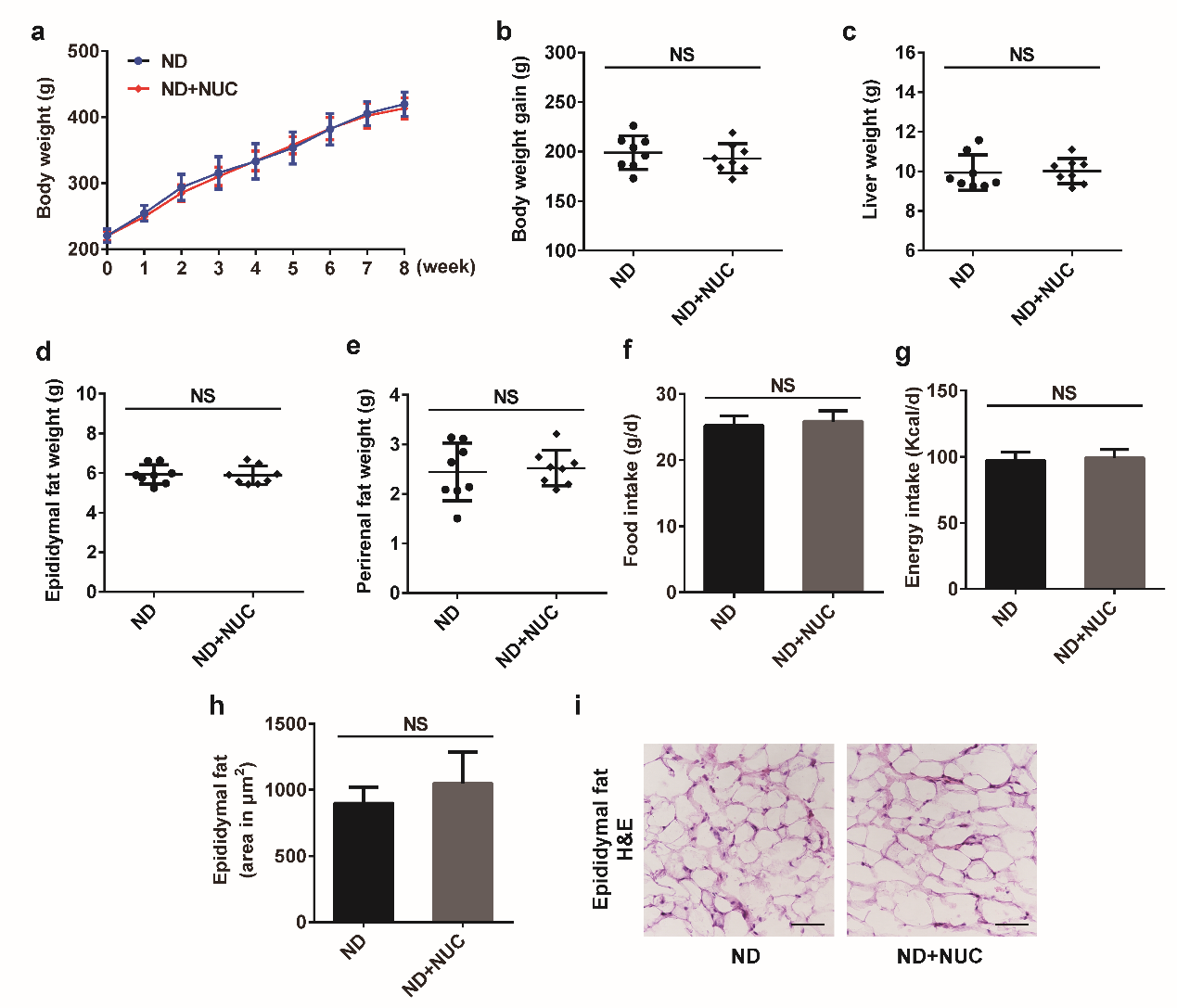


**Figure S1. Effects of NUC on body weight and fat accumulation in ND-fed rats.** The effects of NUC treatment on body weight (a), body weight gain (b), liver weight (c), epididymal fat weight (d), and perirenal fat weight (e) were shown. Food intake (f) and energy intake (g) was monitored. Energy intake was determined based on calorie intake from consumed food. Epididymal fat size (h) was demonstrated using H&E staining (Magnification = 200 ×, scale bar, 50 μm). Mean epididymal fat size (i) was estimated using the Image J software. Values were presented as mean ± SD (n = 8 per group). NS, not significant.


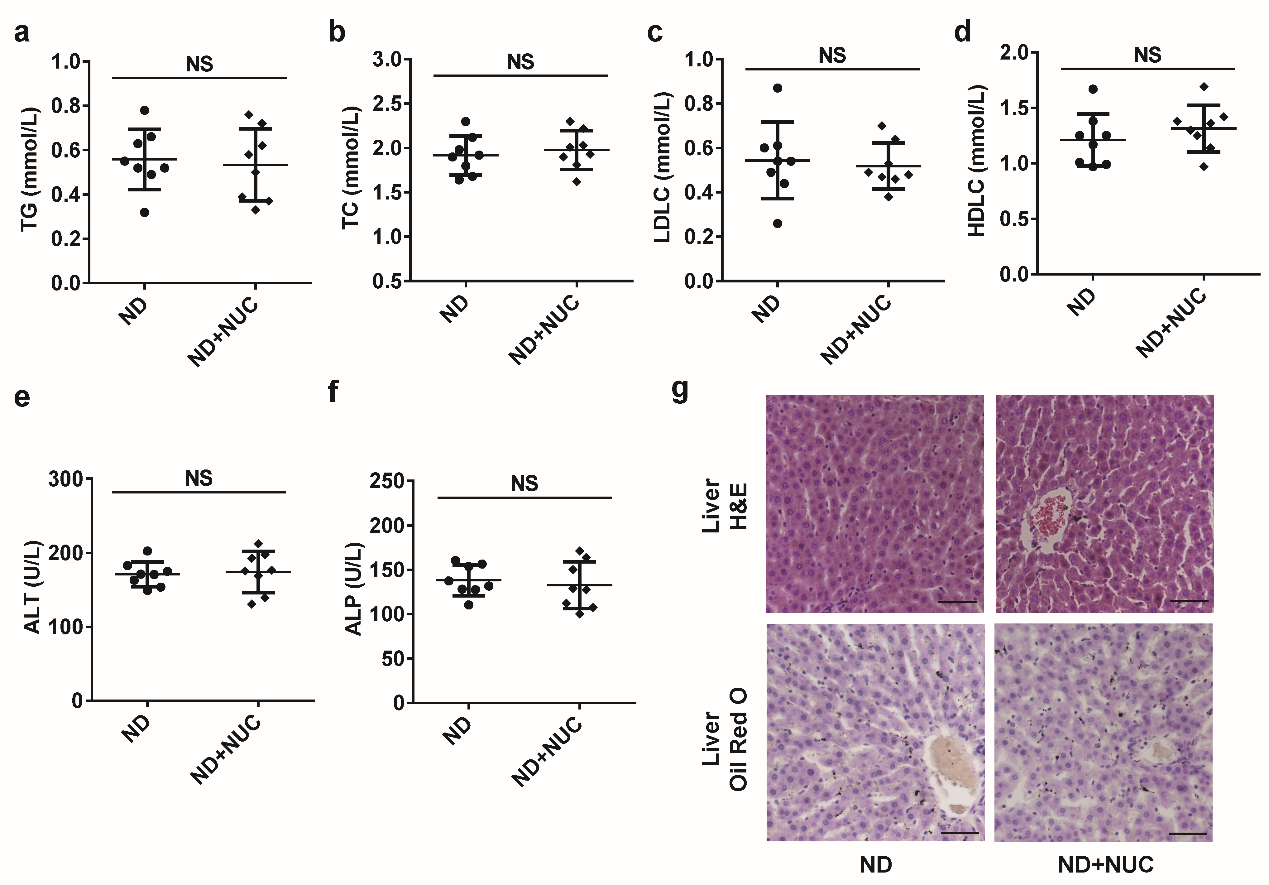


**Figure S2. Effects of NUC on lipid profile, liver function and hepatic fatty deposition in ND-fed rats.** Serum levels of TG (a), TC (b), LDL-C (c), HDL-C (d), ALT (e), and ALP (f) were determined. Liver lipid content (g) was assessed using H&E staining (Magnification = 200 ×, scale bar, 50 μm) and Oil Red O staining (Magnification = 200 ×, scale bar, 50 μm). Values were presented as mean ± SD (n = 8 per group). NS, not significant.


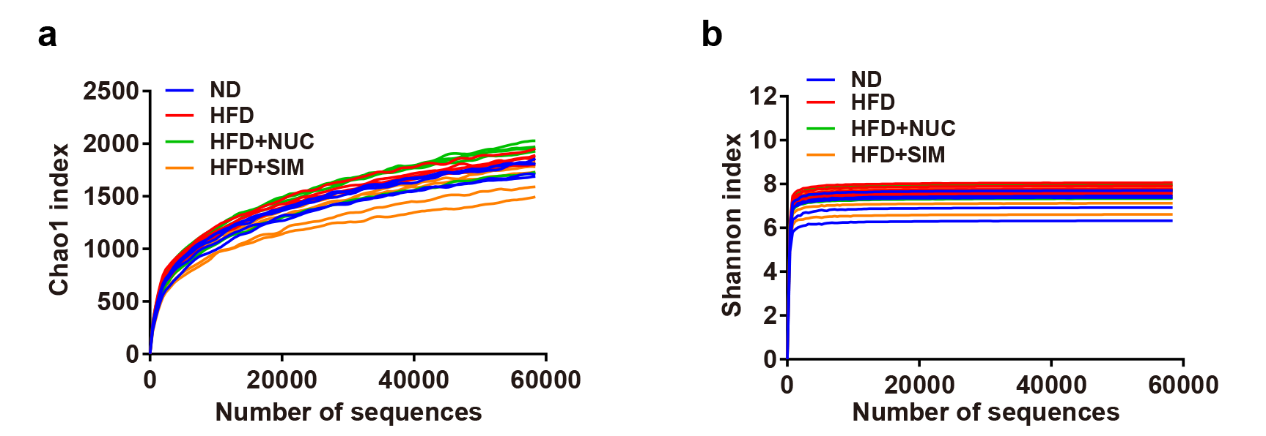


**Figure S3. Alpha diversity analysis of NUC-treated microbiota.** Chao1 (a) and Shannon (b) indices exhibited the diversity and richness of the gut microbiota in rats (n = 5 per group). Each bar represents one rat sample.

**
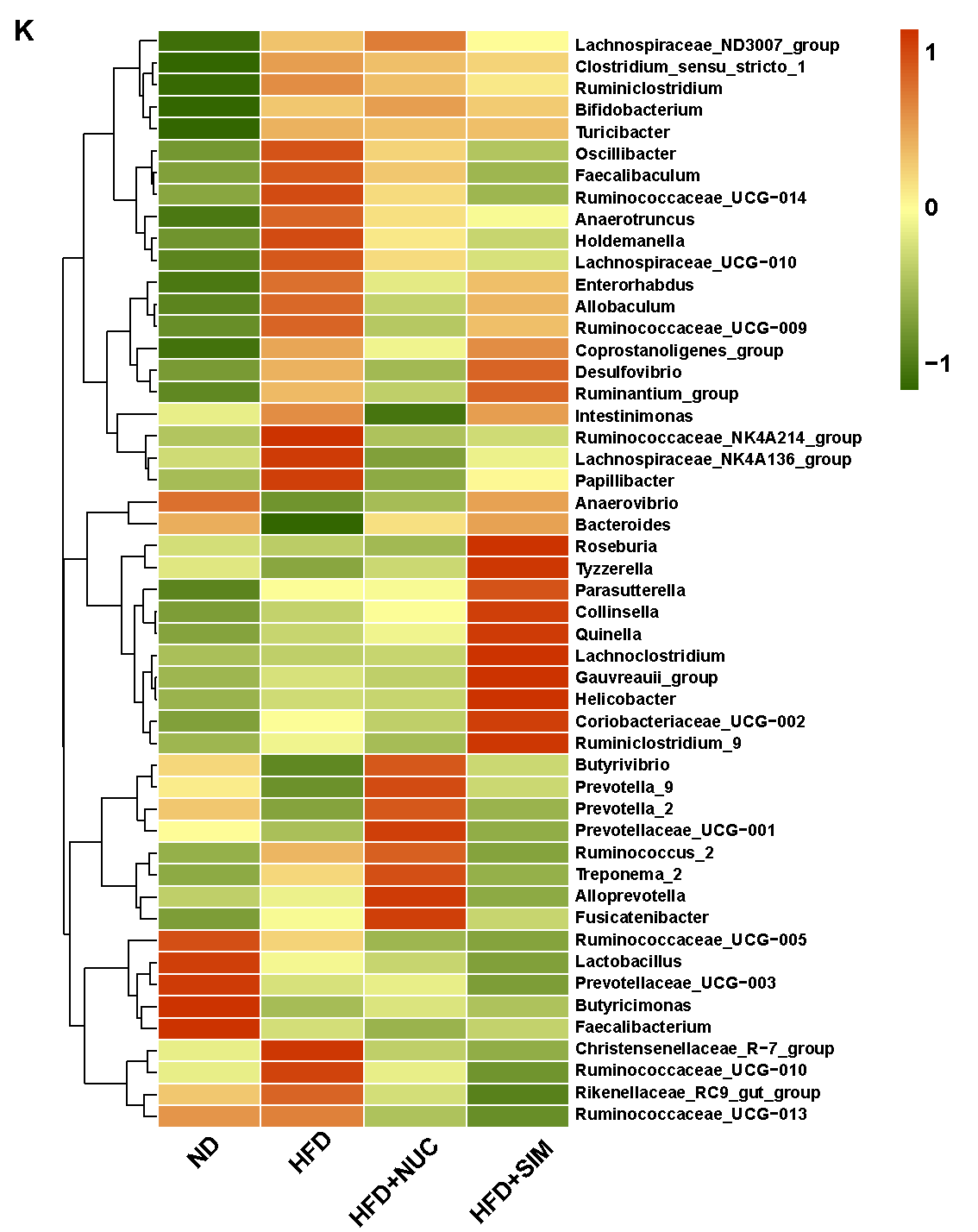
**

**Figure S4. Heat map for the effect of NUC supplementation on the abundance of gut microbiota in HFD-fed rats.** Heat map of the abundance of 50 key genera indicated genus-level changes among the groups (n = 5 per group).


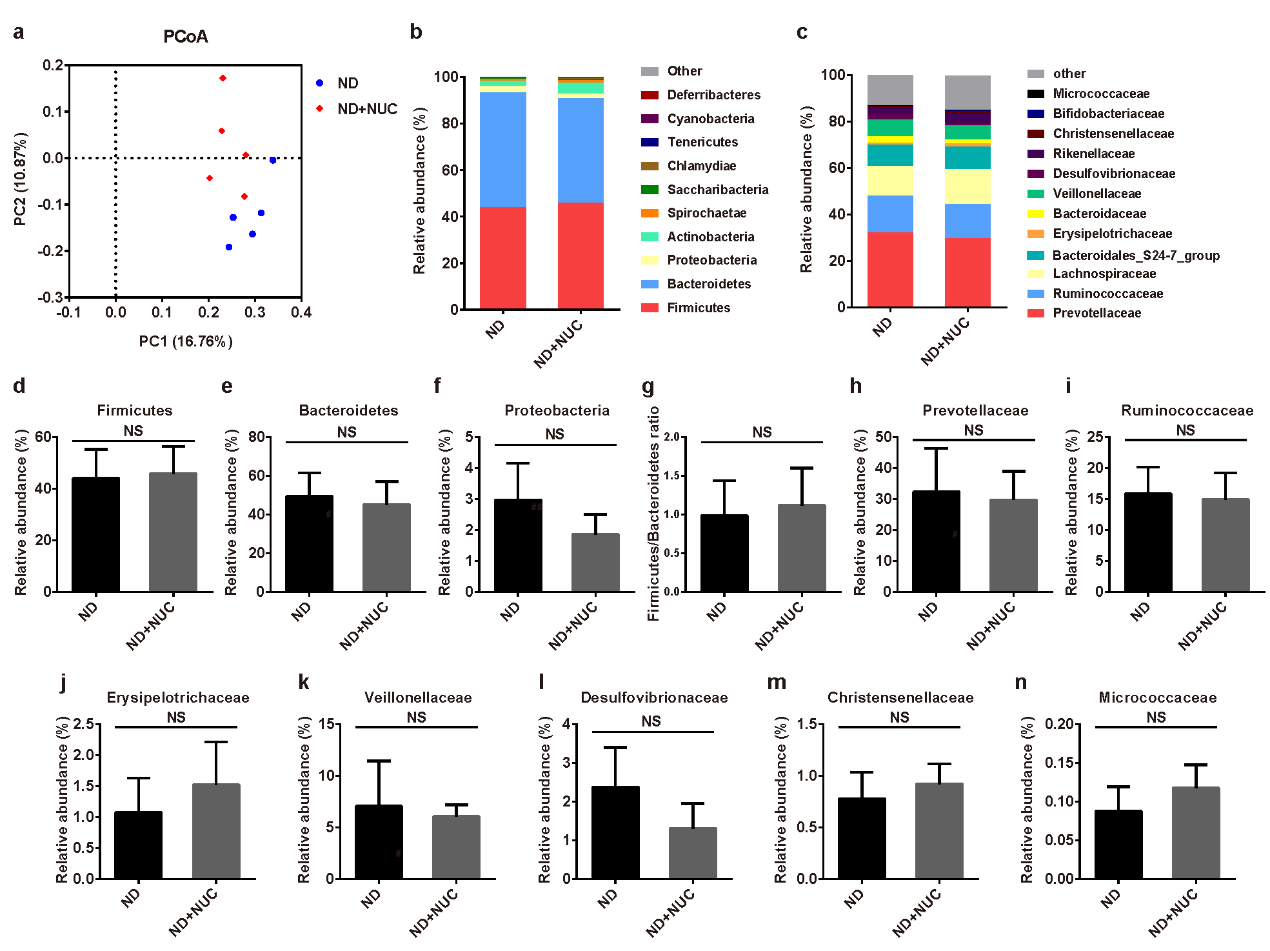


**Figure S5. Effects of NUC on the diversity and composition of gut microbiota in ND-fed rats.** (a) Unweighted UniFrac PCoA plot based on the OTUs abundance of each rat. Bacterial taxonomic profiling in the phylum (b) and family (c) levels of intestinal bacteria from different groups. The relative abundance of the bacterial phyla (e-g) and family (h-n) changes in the fecal samples from different groups. Values were presented as mean ± SD (n = 5 per group). NS, not significant.


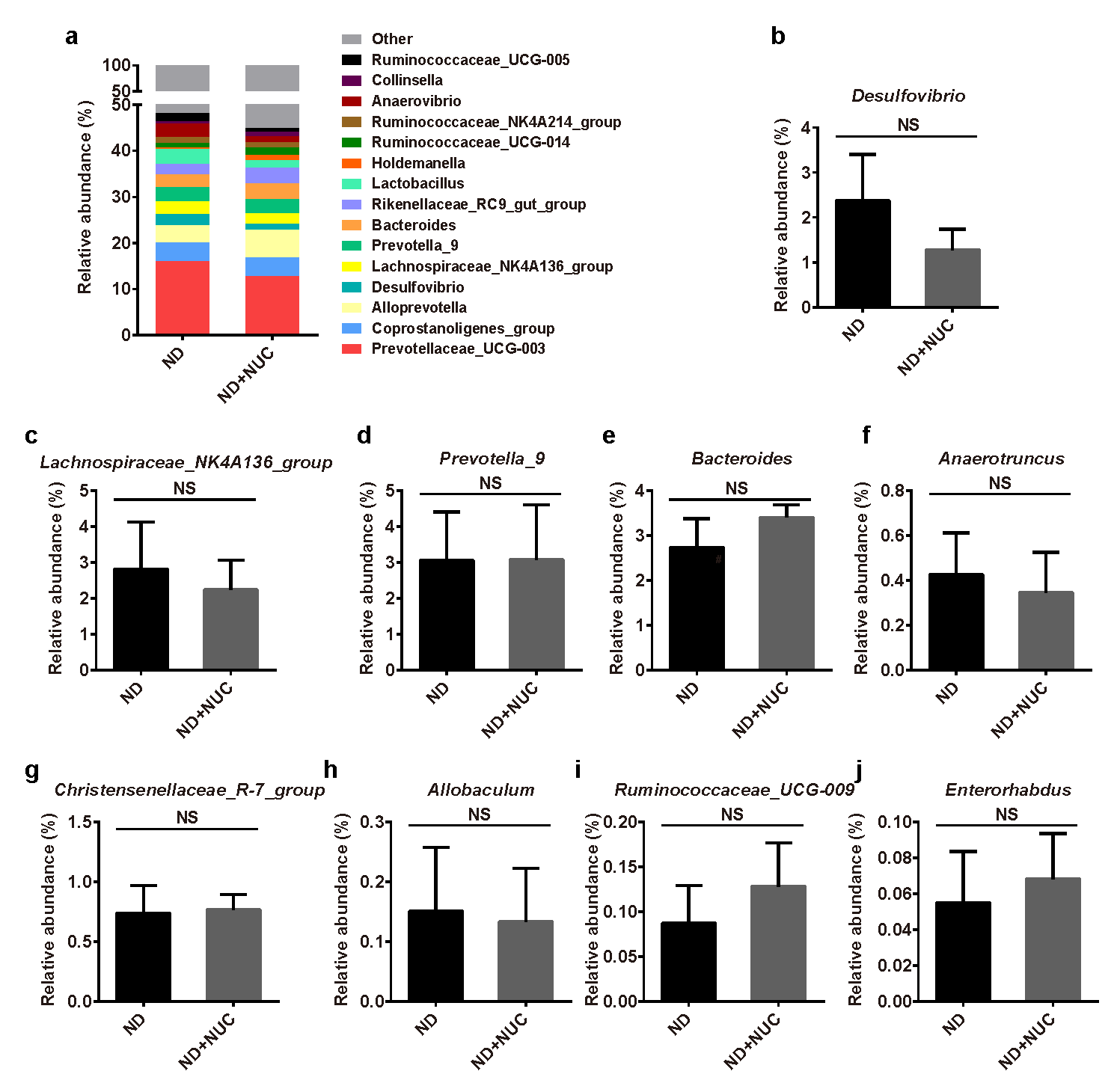


**Figure S6.** **Effects of oral NUC on the abundance of gut microbiota in ND-fed rats**. Bacterial taxonomic profiling in the genus level of intestinal bacteria from different groups (a) was shown. The relative abundances of *Desulfovibrio* (b), *Lachnospiraceae_NK4A136_group* (c), *Prevotella_9* (d), *Bacteroides* (e), *Anaerotruncus* (f), *Christensenellaceae_R-7_group* (g), *Allobaculum* (h), *Ruminococcaceae_UCG-009* (i), and *Enterorhabdus* (j) were shown. Values were presented as mean ± SD (n = 5 per group). NS, not significant.


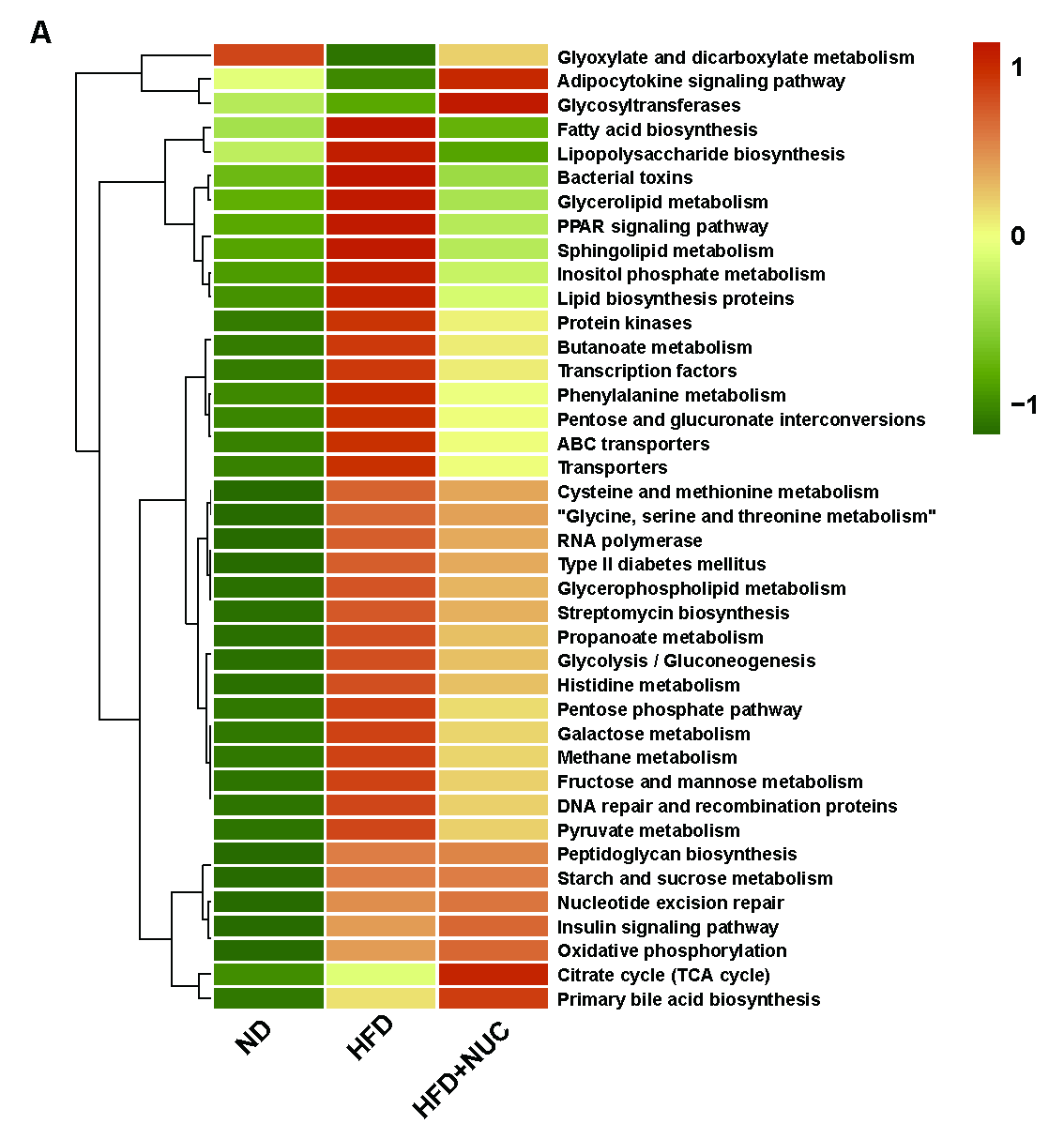


**Figure S7. Heat map for the effect of NUC supplementation on functional potential of gut microbiome in HFD-fed rats.** Metabolic pathways from KEGG module predictions using 16S rRNA data with PICRUSt (n = 5 per group).
